# Predicting three-dimensional genome organization with chromatin states

**DOI:** 10.1371/journal.pcbi.1007024

**Published:** 2019-06-10

**Authors:** Yifeng Qi, Bin Zhang

**Affiliations:** Departments of Chemistry, Massachusetts Institute of Technology, Cambridge, Massachusetts, United States of America; Carnegie Mellon University, UNITED STATES

## Abstract

We introduce a computational model to simulate chromatin structure and dynamics. Starting from one-dimensional genomics and epigenomics data that are available for hundreds of cell types, this model enables *de novo* prediction of chromatin structures at five-kilo-base resolution. Simulated chromatin structures recapitulate known features of genome organization, including the formation of chromatin loops, topologically associating domains (TADs) and compartments, and are in quantitative agreement with chromosome conformation capture experiments and super-resolution microscopy measurements. Detailed characterization of the predicted structural ensemble reveals the dynamical flexibility of chromatin loops and the presence of cross-talk among neighboring TADs. Analysis of the model’s energy function uncovers distinct mechanisms for chromatin folding at various length scales and suggests a need to go beyond simple A/B compartment types to predict specific contacts between regulatory elements using polymer simulations.

## Author summary

Three-dimensional genome organization is expected to play crucial roles in regulating gene expression and establishing cell fate, and has inspired the development of numerous innovative experimental techniques for its characterization. Though significant progress has been made, it remains challenging to construct chromosome structures at high resolution. Following the maximum entropy approach pioneered by Zhang and Wolynes, we developed a predictive model and parameterized a force field to study chromatin structure and dynamics using genome-wide chromosome conformation capture data (Hi-C). Starting from one-dimensional sequence information that includes histone modification profiles and CTCF binding sites, this model predicts chromosome structure at a 5kb resolution, thus establishing a sequence-structure relationship for the genome. A significant advantage of this model over comparable approaches is its ability to study long-range specific contacts between promoters and enhancers, in addition to building high-resolution structures for loops, TADs and compartments. Furthermore, the model is shown to be transferable across chromosomes and cell types, thus opens up the opportunity to carry out *de novo* prediction of genome organization for hundreds of cell types with available epigenomics but not Hi-C data.

## Introduction

The human genome contains about 2 meters of DNA that is packaged as chromatin inside a nucleus of only 10 micrometers in diameter [1]. The way in which chromatin is organized in the three-dimensional space, i.e., the chromatin structure, has been shown to play important roles for all DNA-templated processes, including gene transcription, gene regulation, DNA replication, etc [2–4]. A detailed characterization of chromatin structure and the physical principles that lead to its establishment will thus greatly improve our understanding of these molecular processes.

The importance of chromatin organization has inspired the development of a variety of experimental techniques for its characterization. For example, using a combination of nuclear proximity ligation and high-throughput sequencing, chromosome conformation capture and related methods quantify the interaction frequency in three-dimensional space between pairs of genomic loci [5,6], and have revealed many conserved features of chromatin organization. A consistent picture that is emerging from these experiments is the formation of chromatin loops and topologically associating domains (TADs) at the intermediate scale of kilobases to megabases, and the compartmentalization of chromatin domains that are millions of base pairs apart in sequence [7–11]. Many of the findings from these cross-linking experiments are now being validated and confirmed with microscopy imaging studies that directly probe spatial contacts [12–20].

Polymer modeling has played a critical role in our understanding of the genome organization and in interpreting features of Hi-C contact maps [21]. In particular, due to its deviation from the value of an equilibrium globule [6], the power-law exponent of the contact probability between pairs of genomic segments as a function of the genomic separation has attracted the attention of numerous research groups [22–28]. Of the many mechanisms that have been proposed, the non-equilibrium extrusion model [29–31], which assumes that cohesin molecules function as active enzymes to inch along the DNA and fold the chromatin until encountering bound CTCF molecules, has gained wide popularity [32]. Notably, this model succeeds in explaining the flanking of CCCTC-binding factor (CTCF) and cohesin binding sites at the boundaries of chromatin loops and TADs [7,9–11,33]. On the other hand, phase separation, which is emerging as the key mechanism for organizing numerous membraneless organelles [34–36], has been suggested as the driving force for chromosome compartmentalization [37–39]. Since polymer molecules that differ in chemical compositions are known not to intermix [40], micro-phase separation can contribute to the formation and compartmentalization of chromatin domains with distinct histone modification profiles. Finally, besides these mechanism-based modeling strategies, data-driven approaches have also been quite successful in reconstructing chromosome structures directly from Hi-C data and revealing structural features of both interphase and metaphase chromosomes [41–45].

In parallel, bioinformatics studies have provided powerful tools in addressing potential biases in Hi-C data [46–48], and offered numerous insights in our understanding of genome organization. In particular, correlating one-dimensional genomics and epigenomics data with 3D contacts has been rather informative and has led to many proposals on the molecular mechanism of chromatin folding [4,49–54]. Furthermore, using advanced machine learning techniques, numerous groups have developed predictive models to identify specific contacts between regulatory elements [55–58]. Though not able to construct the whole contact map and 3D chromosome structures, these machine learning approaches have achieved the level of resolution and specificity needed to study functionally important contacts within a TAD. On the other hand, it remains challenging to quantitatively study such functionally important contacts using polymer modeling approaches, though significant progress towards that direction is being made [39,59–63]

The difficulty in predicting contacts between specific regulatory elements using polymer models is at least twofold. First, existing phase separation models based on A/B compartments or six subcompartments are inadequate for such purposes, despite their success in recapitulating the long-range block-wise patterns observed in Hi-C. As chromosome compartments are defined based on contact patterns revealed by Hi-C at a coarse resolution from 50kb to 1 Mb, they tend to group many regulatory elements together as one “active” type and fail to capture the distinction among them [6,7,47]. The ambiguity of these compartments significantly limits the accuracy of polymer models built upon them. To study enhancer-promoter interactions, one must introduce new chromatin types at a higher resolution to achieve the required specificity. How to define these types and how many types are needed remain unclear. Secondly, even with our current understanding of chromatin folding mechanisms, developing a quantitative polymer model to predict contact probability between pairs of genomic loci is still a non-trivial task. In particular, robust and efficient schemes are needed to derive parameters of polymer models to ensure their accuracy.

In this paper, we report the development of a predictive and transferable polymer model to simulate the structure and dynamics of chromosomes at five kilo base resolution. This model takes combinatorial patterns of epigenetic marks and genomic location and orientation of CTCF binding sites as input, and can be parameterized from Hi-C data with a robust and efficient maximum entropy approach [64,65]. A key innovation of this model is its use of chromatin states to capture the wide variety of regulatory elements and to probe their interactions. Computer simulations of this model provide a high-resolution structural characterization of chromatin loops, TADs, and compartments, and succeed in quantitatively reproducing contact probabilities and power-law scaling of 3D contacts as measured in Hi-C and super-resolution imaging experiments. Many significant enhancer-promoter contacts can be captured in simulated contact maps as well. As the model incorporates ingredients from both the extrusion and the phase separation mechanism, its success in quantitative predictions of genome organization provides strong support for such mechanisms. In the meantime, detailed analysis of the model parameters further reveals a significant difference between the interactions that stabilize TAD and those that drive compartmentalization, providing additional insight into chromatin folding not appreciated in existing modeling efforts. Finally, we demonstrate that the model is transferable across chromosomes and cell types, setting the stage for *de novo* prediction of the structural ensemble for any given chromatin segment using only one-dimensional sequencing data that is available for hundreds of cell types.

## Results

### Predictive modeling of chromatin organization

**Fig 1:**
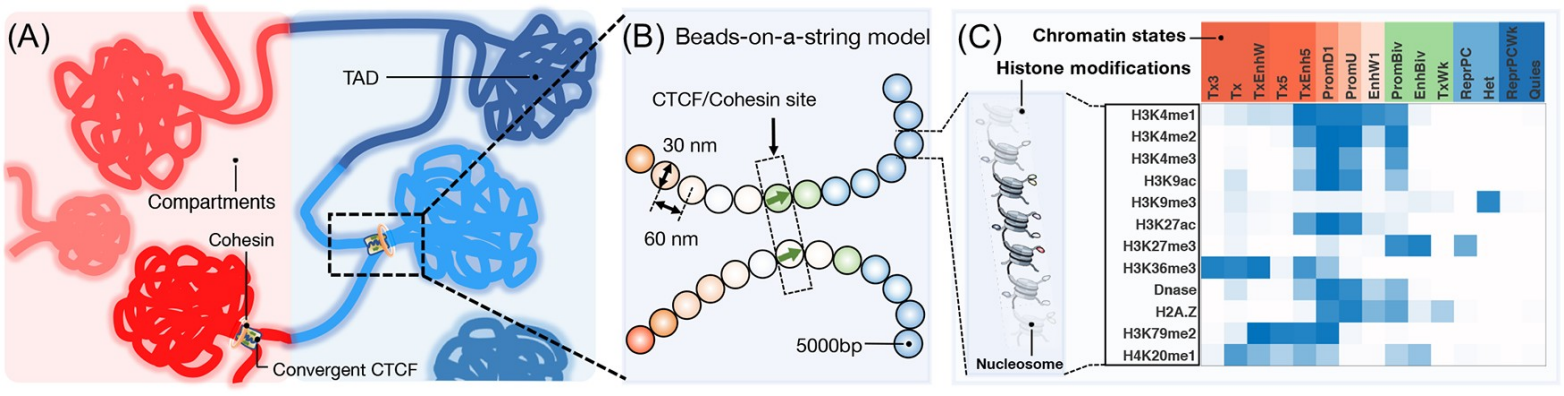
Overview of the key elements of the computational model. (A) Illustration of genome organization at various length scales that includes the formation of CTCF mediated chromatin loops, TADs, and compartments. (B) A schematic representation of the computational model that highlights the assignment of chromatin states and CTCF binding sites. Chromatin states for each bead—a 5kb long genomic segment—are derived from the combinatorial patterns of histone marks. They are shown in part (C) as a heat map with darker colors indicating higher probabilities of observing various marks.

We introduce a predictive model to study cell-type specific 3D chromatin folding. This model takes a sequence of chromatin states derived from genome-wide histone modification profiles and a list of CTCF binding sites as input. We selected these genomic features due to their known roles in organizing the chromatin at various length scales (Fig 1A). At the core of this model is an energy function—a force field—that is sequence specific and ranks the stability of different chromatin conformations. Starting from the input for a given chromatin segment, we use molecular dynamics simulations to explore chromatin conformations dictated by the energy function and to predict an ensemble of high-resolution structures. These structures can be compared directly with super-resolution imaging experiments or converted into contact probability maps for validation against genome-wide chromosome conformation capture (Hi-C) experiments.

As shown in Fig 1B, a continuous genomic segment is represented as beads on a string in this model. Each bead accounts for five-kilo bases in sequence length and is assigned with a chromatin state derived from the underlying combinatorial patterns of 12 key histone marks. Chromatin states are known to be highly correlated with Hi-C compartment types [39,54,66] and, therefore, will help model large-scale chromosome compartmentalization. In the meantime, chromatin states can go beyond traditional A/B compartments or subcompartments to provide polymer models with the specificity needed for studying interactions between regulatory elements. We define a total of 15 chromatin states, identified using a hidden Markov model [67], to distinguish promoters, enhancers, heterochromatin, quiescent chromatin, etc (see Methods). Detailed histone modification patterns for these chromatin states are shown in Fig 1C. We note that 15 is large enough to capture the diversity of epigenetic modifications while still being small enough to ensure a sufficient population of each state for a robust inference of interaction parameters between them (Figure A1 in S1 Supporting Information). We further studied a hidden Markov model with 20 states, and found that further increasing the number of states does not lead to a discovery of additional epigenetic classes with significant populations (Figure A2 in S1 Supporting Information). A polymer bead is further labeled as a CTCF site to mark chromatin loop boundaries if both CTCF and cohesin molecules are found to be present in the corresponding genomic region. We define the orientation of these CTCF sites by analyzing the underlying CTCF motif and the relative position of CTCF molecules with respect to cohesin. Details for the definition of CTCF binding sites are provided in **Methods**.

The potential energy for a given chromatin configuration ***r*** is a sum of three components, and *U*_Chrom_(***r***) = *U*(***r***) + *U*_CS_(***r***) + *U*_CTCF_(***r***). *U*(***r***) is a generic polymer potential that is included to ensure the continuity of the chromatin, and to enforce excluded volume effect among genomic loci. *U*_CS_(***r***) is a key innovation of the chromatin model, and is crucial to capture the formation of TADs and compartments. It quantifies the chromatin state specific interaction energies between pairs of loci. As detailed in *Section*: *Physical principles of chromatin organization* and **Methods**, we used a general form for *U*_CS_(***r***) to capture its dependence on genomic separation. *U*_CTCF_(***r***) is inspired by the loop extrusion model [29–31], and facilitates the formation of loop domains enclosed by pairs of CTCF binding sites in convergent orientation (Fig 1A). Both *U*_CS_(***r***) and *U*_CTCF_(***r***) contain adjustable parameters that can be derived from Hi-C data following the optimization procedure developed by one of the authors [64,65]. Segments of chromosomes 1, 10, 19 and 21 from GM12878 cells were used for parameterization to ensure a sufficient coverage of all chromatin states (see Figure A1 in S1 Supporting Information). Detailed expressions for the potential energy, and the parameterization procedure are provided in **Methods** and in the S1 Supporting Information.

**Fig 2:**
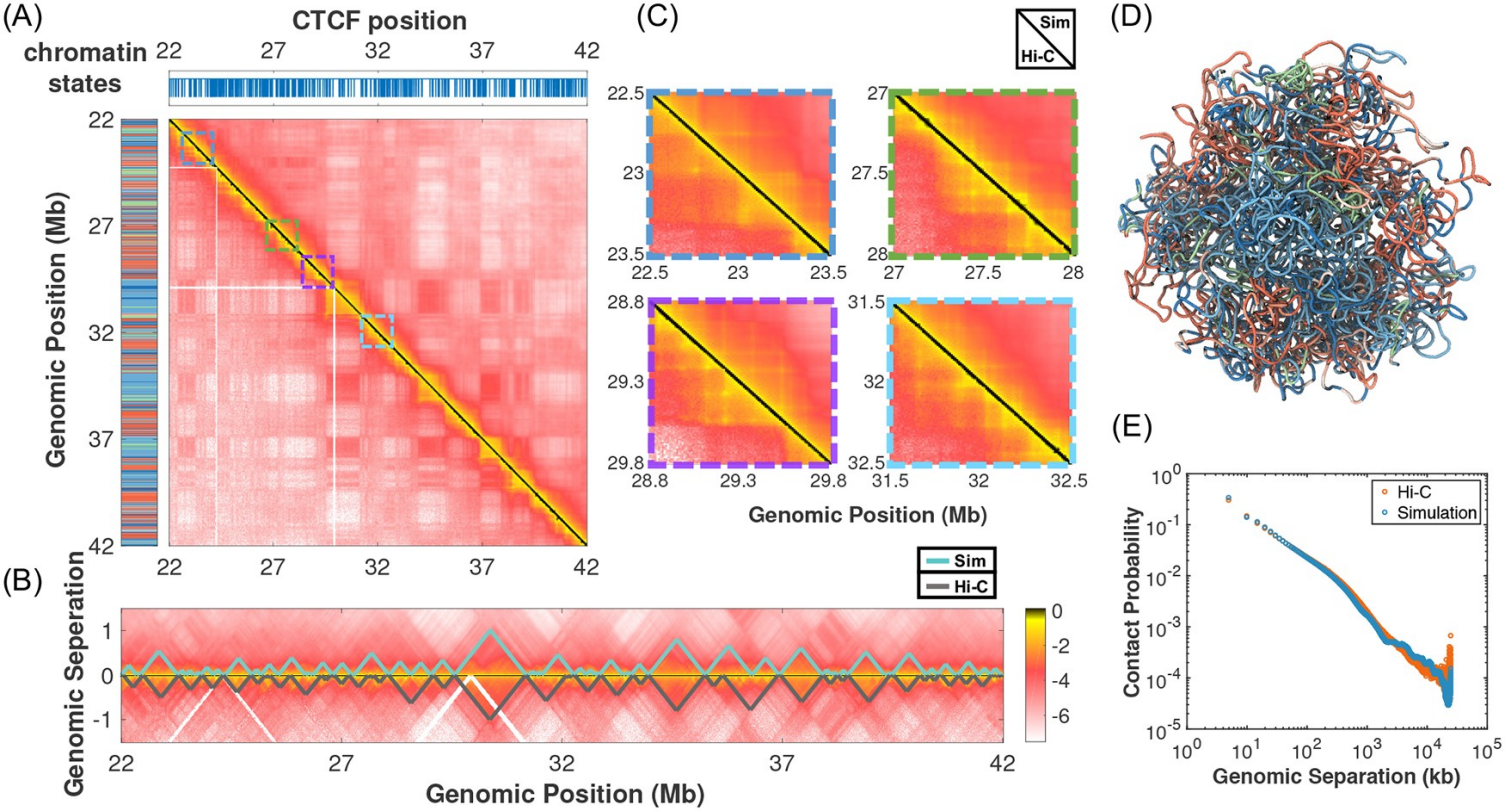
Comparison between simulated and experimental contact probability maps for a 20 Mb segment of chromosome 1 from GM12878 cells. (A) Results from simulation and the Hi-C experiment performed in Ref. [7] are shown in the upper and lower triangle respectively on a log scale. Also shown on the left and top panels are the sequence of chromatin states and the genomic positions of CTCF binding sites. (B) A zoomed-in view of the contact maps along the diagonal region to highlight the formation of TADs. TAD boundaries detected using the software TADbit are plotted on the top of the contact map, with the simulation shown in cyan and experiment in grey. (C) Zoomed-in view of several representative regions along the diagonal to highlight the formation of chromatin loops. (D) A representative chromatin structure predicted by the computational model is drawn in a tube representation and colored by chromatin states. (E) The average contact probability as a function of the genomic separation is shown below on a log-log scale for the simulated (blue) and experimental (red) contact maps respectively.

Using the parameterized energy function, we simulated the ensemble of chromatin structures and determined the corresponding contact probability map for a 20 Mb region of chromosome 1 from GM12878 cells. As shown in Fig 2A, the simulated contact map is in good agreement with the one measured by Hi-C experiments from Ref. [7] and reproduces the overall block-wise checkerboard pattern that corresponds to the compartmentalization of chromatin domains. A zoomed-in view along the diagonal of the contact map provided in Fig 2B and 2C further suggests that chromatin TADs and loops are also well reproduced. Similar comparisons for other chromosomes used in parameterizing the model are provided in Figure B in S1 Supporting Information. We note that the length 20 Mb was chosen for computational efficiency, but the model can be easily generalized to longer chromatin segments (see Figure C in S1 Supporting Information).

To go beyond the visual inspection and quantify the correlation between simulated (GM-Sim) and experimental (GM-Exp) contact maps, we calculated the Pearson correlation coefficient (PCC) between the two for chromosome 1 and found that it exceeds 0.96. Importantly, this number is higher than the PCC (0.94) between GM-Sim and Hi-C data from IMR90 cells (IMR-Exp). Breaking down the PCC at different genomic separations also supports that GM-Sim is more correlated with GM-Exp at all ranges than with IMR-Exp (Figure D in S1 Supporting Information). In addition, we also determined the stratum-adjusted correlation coefficient (SCC) that takes into account the distance-dependence effect of contact maps by stratifying them according to the genomic distance [68], and obtained 0.7 for GM-Sim/GM-Exp, and 0.66 for GM-Sim/IMR-Exp. Therefore, SCC analysis also validates our model’s ability in reproducing Hi-C contact maps and in capturing the distinction between cell types. We note that the magnitude of SCC can be sensitive to the smoothing parameter used in its calculation and should be interpreted with caution (Figure E in S1 Supporting Information).

We further examined the agreement between simulated and experimental contact maps using multiple feature-specific metrics. First, we define the contact enhancement for a pair of genomic loci as the ratio of their contact probabilities over the mean contacts averaged over a locally selected background region (see Figure F1 in S1 Supporting Information). The contact enhancement for chromatin loops from chromosome 1 is always larger than one, indicating a strong enhancement of spatial colocalization between loop anchors. Furthermore, over 74% of the loop pairs exhibit a contact enhancement that is larger than the 90th percentile of the distribution for random genomic pairs. These random pairs are selected regardless of CTCF occupancy but with comparable sequence separations as those found in chromatin loops. Therefore, if we use the 90th percentile of the random distribution as a threshold (1.16) and predict every convergent CTCF pairs as loops, the prediction will have a false negative rate of 26%, and a false positive rate less than 10%. The false positive value is an upper bound since most of the random pairs are not flanked with convergent CTCF. The sensitivity of chromatin loop predictions on the threshold is shown in Figure F2 in S1 Supporting Information. It is worth pointing out that the contact enhancement for chromatin loops calculated using Hi-C data is in general larger than simulated values and separates better from that for random pairs (Figure F3 in S1 Supporting Information). The overlap between the two distributions in our simulation is due to that random pairs include a significant fraction of convergent CTCF pairs whose contacts are enhanced as a result of the potential *U*_CTCF_(***r***). Many of these pairs, however, are not recognized as loops in Hi-C, and more advanced algorithms than simple binding site orientations are probably needed to identify loop forming CTCF pairs [69].

To go beyond CTCF mediated contacts and evaluate our model’s ability in reproducing strong interactions between genomic loci, we selected statistically significant contact pairs from simulated and experimental contact maps for chromosome 1 using the software Fit-Hi-C [48] (Figure G in S1 Supporting Information). As a quantitative metric, we define the matching score as the percent of experimental pairs that can be found in the list extracted from simulation. The reverse matching score can be similarly defined as the percent of simulated pairs found in the experimental list. The matching score for the top 1000 chromatin contacts is determined to be 46% and 52% for the reverse matching. To examine specific interactions between regulatory elements, we performed a similar analysis by selecting the top 100 enhancer (state: EnhW1)-promoter (state: PromD1) pairs with highest contact probabilities based on simulated and experimental contact maps. We find that over 70% of experimental pairs are captured in our simulation for chromosome 1. These results suggest that our model based on chromatin states and CTCF mediate interactions is able to reproduce a large fraction of significant contacts detected in Hi-C experiments. Further improving the model’s ability in predicting functionally important pairs would potentially require considering the effect of other proteins, such as YY1 that are known to mediate chromatin interactions [70], and will be an interesting future direction.

We next determined the correlation coefficients between the top five eigenvectors for simulated and experimental contact matrices. As shown in Figure H in S1 Supporting Information, the contact maps reconstructed using only these eigenvectors recapitulate the formation of TADs and compartments observed in the original maps. The high correlation between simulated and experimental eigenvectors (with PCC at approximately 0.8) supports that the corresponding features are well captured by the computational model, and confirms the qualitative observations from Fig 2 and Figure B in S1 Supporting Information.

To more closely examine the quality of simulated TADs, we calculated the insulation profile by sliding a uniform 500kb × 500kb square along the diagonal of the contact matrix and averaging over all contacts within the square. The minima of this profile can be used to identify TAD boundaries as inter-TAD contacts are sparser compared to intra-TAD contacts, resulting in a drop in the insulation score profile as the sliding window crosses TAD boundaries [71]. The PCC between experimental and simulated insulation profiles for chromosome 1 is 0.7. We find that the matching score for TAD boundaries is 80% and 100% for the reverse matching. As another independent validation, we determined TAD boundaries using the software TADbit [43], and found that the simulated results again match well with experimental ones (see Figure I in S1 Supporting Information).

**Fig 3:**
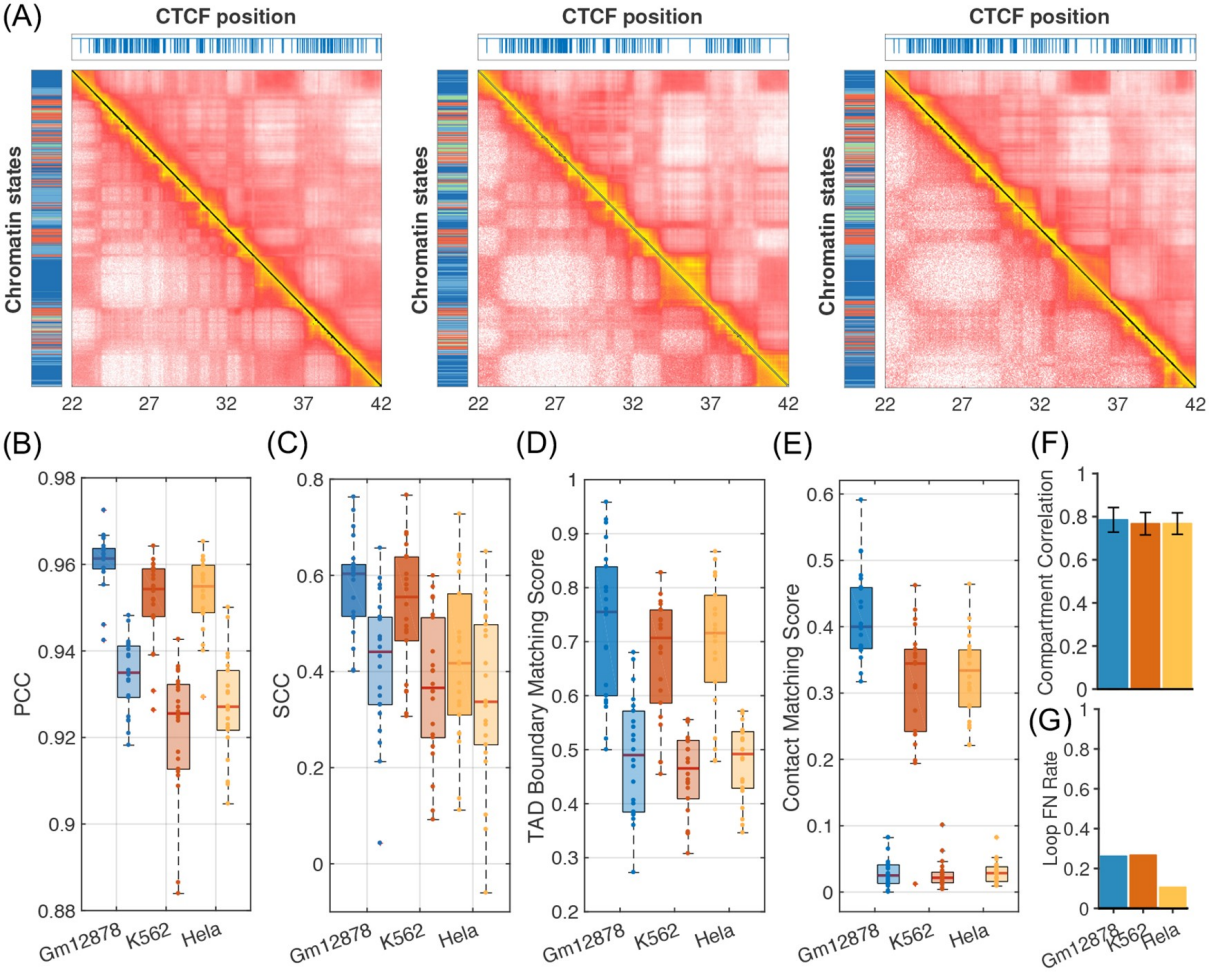
Transferability of the computational model across chromosomes and cell types. (A) Comparison between simulated (*Top right*) and experimental (*Bottom left*) contact maps for chromosome 2 from GM12878 (*Left*), K562 (*Middle*), and Hela cells (*Right*). (B-E) Quality of computational predictions for all chromosomes from the three cell types measured by Pearson (PCC) and stratum-adjusted correlation coefficients (SCC) between simulated and experimental contact maps (B,C), matching score for TAD boundaries detected from insulation profiles (D), and matching score for the top 1000 significant contacts (E). Each data point represents one chromosome. Data shown as light colors in (B,C) correspond to PCC/SCC between simulated and IMR90 experimental contact maps, while those in (D,E) correspond to matching scores between experimental and control data. The boxes represent the 25% and 75% quantities of the matching score distribution, and the thick line inside each box corresponds to the median value. Whiskers indicate the last values that fall within 1.5 times the interquartile range. (F) Average correlation coefficients between the top five eigenvectors for the logarithm of contact matrices for all the three cell types. Error bars correspond to standard deviations of the results for all chromosomes. (G) False negative rates for predicting chromatin loops identified in Hi-C data with convergent CTCT binding sites in different cell types.

To demonstrate the transferability of the computational model across chromosomes and cell types, we performed additional simulations for chromosomes from GM12878, K562, and Hela cells, whose Hi-C data were not included during the parameterization procedure. As shown in Fig 3 and Figure J in S1 Supporting Information, these *de novo* predictions are in good agreement with experimental results as measured by PCC (Fig 3B) and SCC (Fig 3C) between experimental and simulated contact maps, matching score between TAD boundaries detected from the insulation profile (Fig 3D) and from TADbit (Figure K1A in S1 Supporting Information), PCC between experimental and simulated insulation profiles (Figure K1D in S1 Supporting Information), matching score between significant contacts detected using Fit-Hi-C (Fig 3E), matching score between interacting enhancer-promoter pairs (Figure K2C in S1 Supporting Information), correlation coefficients of the top five eigenvectors (Fig 3F and Figure H in S1 Supporting Information), and false negative rate of loop predictions (Fig 3F). Furthermore, the model succeeds in revealing the cell-type specificity of Hi-C contact maps, and the simulated contact maps are always more correlated with the corresponding experimental data from the same cell type than with those from IMR90 cells (light colors in Fig 3B and 3C). The matching scores between experimental and simulation results are also significantly higher than those calculated between experimental and control data (light colors in Fig 3D and 3E), which were obtained by randomly shuffling the size of loops/enhancer-promoter pairs/TADs along the chromosome while keeping their total number unchanged. The success of these *de novo* predictions supports that the chromatin-state-based model introduced here provides a consistent description of the 3D genome organization across cell types.

### Structural characterization of chromatin organization

We next analyze the simulated 3D structural ensembles to gain additional insights on chromatin organization. Consistent with previous experimental and theoretical studies [37,72,73], our model reproduces the clustering of active chromatin state and their preferred location at the exterior of chromosomes (Figure L in S1 Supporting Information).

**Fig 4:**
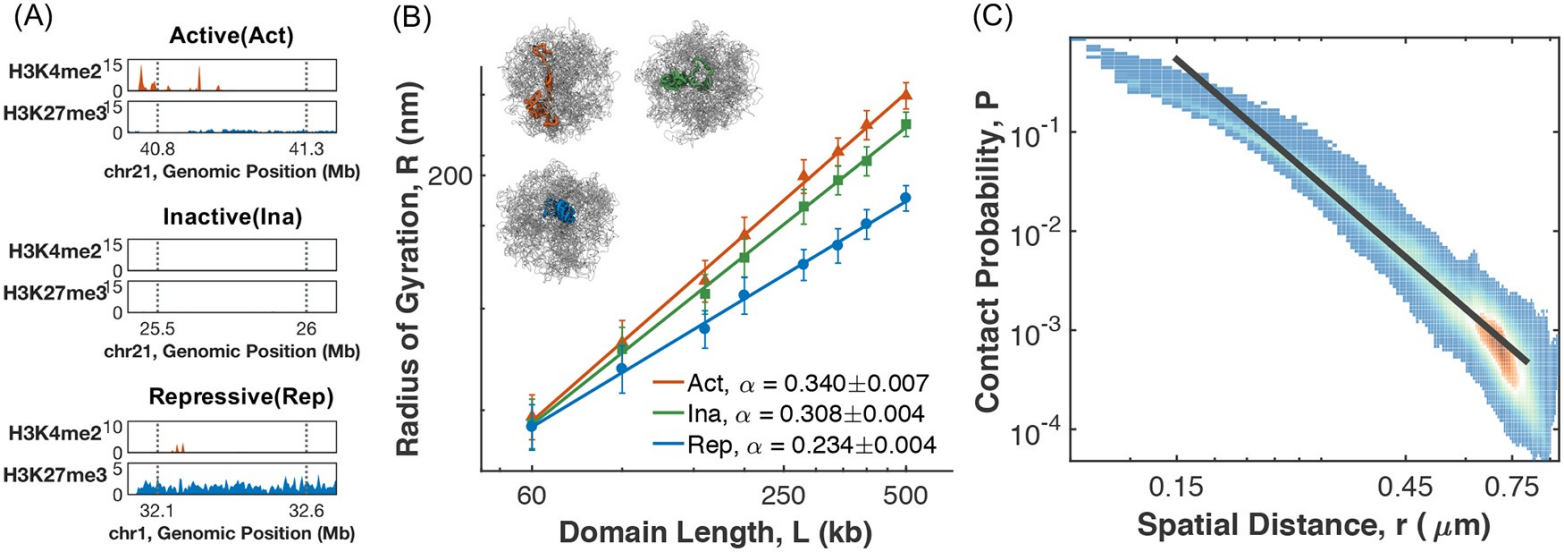
Simulated chromatin structures reproduce findings from super-resolution microscopy experiments. (A) Characteristic histone modification profiles for repressive, active and inactive chromatin. (B) The sizes of repressive (blue), active (orange) and inactive (green) chromatin domains, as measured by their radiuses of gyration, are plotted as a function of the genomic separation on a log scale. The straight lines correspond to numerical fits of the data with a power-law expression *R* = *R*_*o*_*L*^*α*^, with the values of *α* shown in the legend. Representative structures of 500kb in length for the three chromatin types are shown in the inset. Error bars correspond to standard deviations of structures from the entire simulated ensemble. (C) Scatter plot of the contact probabilities between pairs of genomic loci versus their spatial distances shown on a log-log scale. The black line is the best fit to the data using the expression *P* = *P*_*o*_*r*^*β*^, with *β* = −4.18.

Super-resolution imaging experiments probe chromatin organization in 3D space to quantify spatial distances between genomic segments. These 3D measurements can be compared directly with simulated chromatin structures, and thus provide a crucial validation of the computational model parameterized from Hi-C experiments with independent datasets. To understand the overall compactness of various chromatin types, we selected a set of active, repressive and inactive chromatins and determined their radiuses of gyration from the ensemble of simulated structures. These different chromatin types are identified using two key histone marks H3K4me2 and H3K27me3 (Fig 4A). The complete list of chromatin domains with their genomic locations is provided in the Extended Data Sheet. As shown in Fig 4B, the radius of gyration increases at larger genomic separation following a power law behavior in all cases with exponents of 0.34, 0.31 and 0.23 for the three chromatin types respectively. These scaling exponents are in quantitative agreement with imaging measurements performed for Drosophila chromosomes [12] and support the notion that active chromatins adopt less condensed conformations to promote gene activity. Consistent with the imaging study performed on chromosome 21 from IMR90 cells [13,20], we also observe a strong correlation between Hi-C contact probabilities and spatial distances for pairs of genomic loci (Fig 4C).

**Fig 5:**
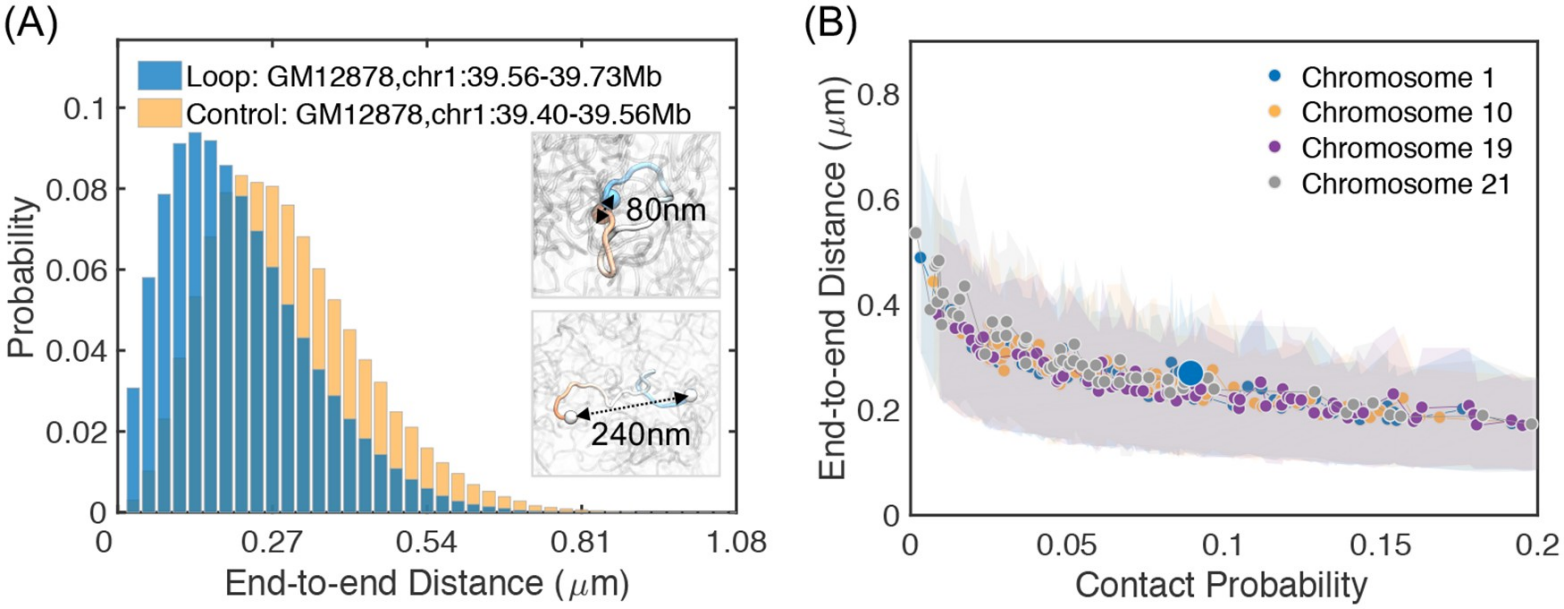
Structural characterization of chromatin loops. (A) Probability distribution of the end-to-end distance for the chromatin loop formed between chr1:39.56 Mb and chr1:39.73 Mb from GM12878 cells (blue) and for a random genomic pair (yellow). Two example configurations that correspond to open and closed chromatin loop structures are shown in the inset. (B) End-to-end distances of chromatin loops versus their corresponding contact probabilities. The shaded areas represent the variances in distances estimated from the simulated structural ensemble.

One of the most striking features revealed by high-resolution Hi-C experiments is the formation of chromatin loops anchored at pairs of convergent CTCF sites [7,10,74,75]. Microscopy studies that directly visualizes 3D distances using fluorescence in situ hybridization (FISH) methods further find that these loops are dynamic, and despite their high contact frequencies, loop anchors are not in close contact in every cell [16,41,76]. Consistent with their dynamic nature, chromatin loops in our simulation adopt flexible conformations as well. As shown in Fig 5A, for the loop formed between chr1:39.56–39.73 Mb, we observe a large variance in the probability distribution of its end-to-end distances. Additional results for other loop pairs are provided in Figure M in S1 Supporting Information. Two example configurations of the loop domain with distance at 0.08 and 0.24 *μm* are shown in the inset. A systematic characterization of all the loops identified in Ref. [7] for the simulated chromatin segment shows that the conformational flexibility is indeed general, though there is a trend in decreasing variance for loops with larger contact probabilities (Fig 5B). We also emphasize that though higher contact probabilities, in general, corresponds to smaller end-to-end distances, their relationship is not strictly monotonic. The opposite correlation can be seen in numerous cases in Fig 5B. Such seemingly paradoxical observations have indeed been found in previous experimental studies that compare 3C with FISH experiment [16,77], and can naturally arise as a result of dynamical looping or loop extrusion [78].

Compared to chromatin loops, TADs are longer and are stabilized by a complex set of interactions [79]. The analysis of their structural ensemble is less straightforward, and the end-to-end distance may not be sufficient for a faithful description of their conformational fluctuation [80]. It is desirable to analyze TAD structures using reaction coordinates that not only help to distinguish different clusters of chromatin conformations, but can also provide insight into the mechanism of TAD folding and formation. Borrowing ideas from protein folding studies, we approximate these reaction coordinates using collective variables with slowest relaxation timescales as determined following the diffusion map analysis [81,82]. Progression along these variables approximates well the most likely transition between two sets of structures and can, therefore, shed light on the pathway for conformational rearrangements. Diffusion map analysis has been successfully applied to a variety of systems to provide mechanistic insights on the conformational dynamics involved in protein folding, ligand diffusion, etc. [83,84].

**Fig 6:**
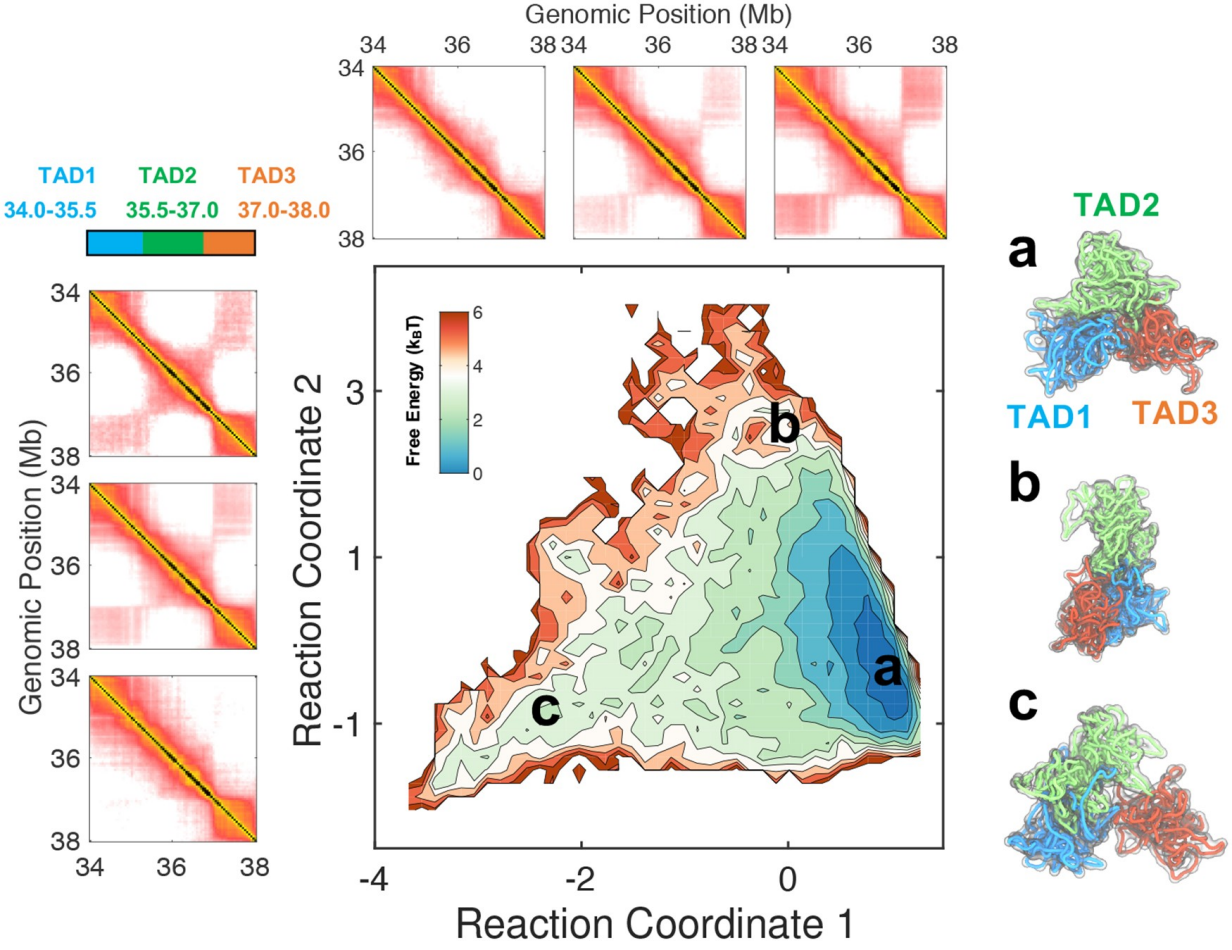
Structural characterization of topologically associating domains using the diffusion map technique. (*Center*) Free energy profile of TAD conformations projected onto two coordinates that describe the slowest collective motions. The (*Left*) and (*Top*) panels illustrate the change in contact maps along the two coordinates. (*Right*) Representative structures for the chromatin segment at various positions indicates in the central and bottom panel. The three contact maps for reaction coordinate 1 were calculated using chromatin structures that fall into the regions [−2.5, −0.5), [−0.5, 0.5) and [0.5,1.5). The three regions used to determine the contact maps for reaction coordinate 2 are [−2.5, −1.0), [−1.0, 1.5), and, [1.5, 3.5).

We applied the diffusion map technique to the predicted structural ensemble of the genomic region chr1:34–38 Mb from GM12878 cells that consists of three visible TADs. As shown in Fig 6, several basins are observed in the probability distribution of chromatin conformations projected onto the first two reaction coordinates, suggesting the presence of multiple stable TAD structures, rather than a unique one. Conformational heterogeneity in TADs has indeed been observed in a recent super-resolution imaging study that characterizes single cell chromatin structures [20]. To gain physical intuition on the reaction coordinates and insight on the transition between TAD structures, we calculated the corresponding contact maps at various values of these coordinates. As shown in the top panel, reaction coordinate one captures the formation of contacts between TAD1 and TAD3 while the structures for all three TADs remain relatively intact. On the other hand, progression along reaction coordinate two (left panel) leads to significant overlaps between TAD1 and TAD2. Interaction between TAD2 and TAD3 can also be observed along a third coordinate as shown in Figure N in S1 Supporting Information. Example structures for the three TADs in various regions are also provided on the right panel. These results are consistent with the notion that TADs are stable structural units for genome organization [79], but also suggest the presence of significant cross-talk among neighboring TADs [85].

### Physical principles of chromatin organization

Though the exact molecular mechanism and driving force for chromatin folding remain elusive, it is becoming increasingly clear that different molecular players are involved in organizing the chromatin at various length scales [49,60,86,87]. For example, transcription factors and architectural proteins are critical in stabilizing the formation of chromatin loops and TADs [4,33,79]. On the other hand, nuclear compartments, such as the nucleolus and the nuclear envelope, contribute to chromatin compartmentalization and mediate contacts among chromatin domains separated by tens of Mb in sequence [50,88]. We expect that these different molecular mechanisms will give rise to distinct interaction energies at various genomic length scales. For example, for the same pair of chromatin states, as the genomic separation between them is varied, the interaction energy that stabilizes their contact should vary. In the following, we examine the dependence of inferred contact energies on genomic separation to reveal the principles of genome organization.

**Fig 7:**
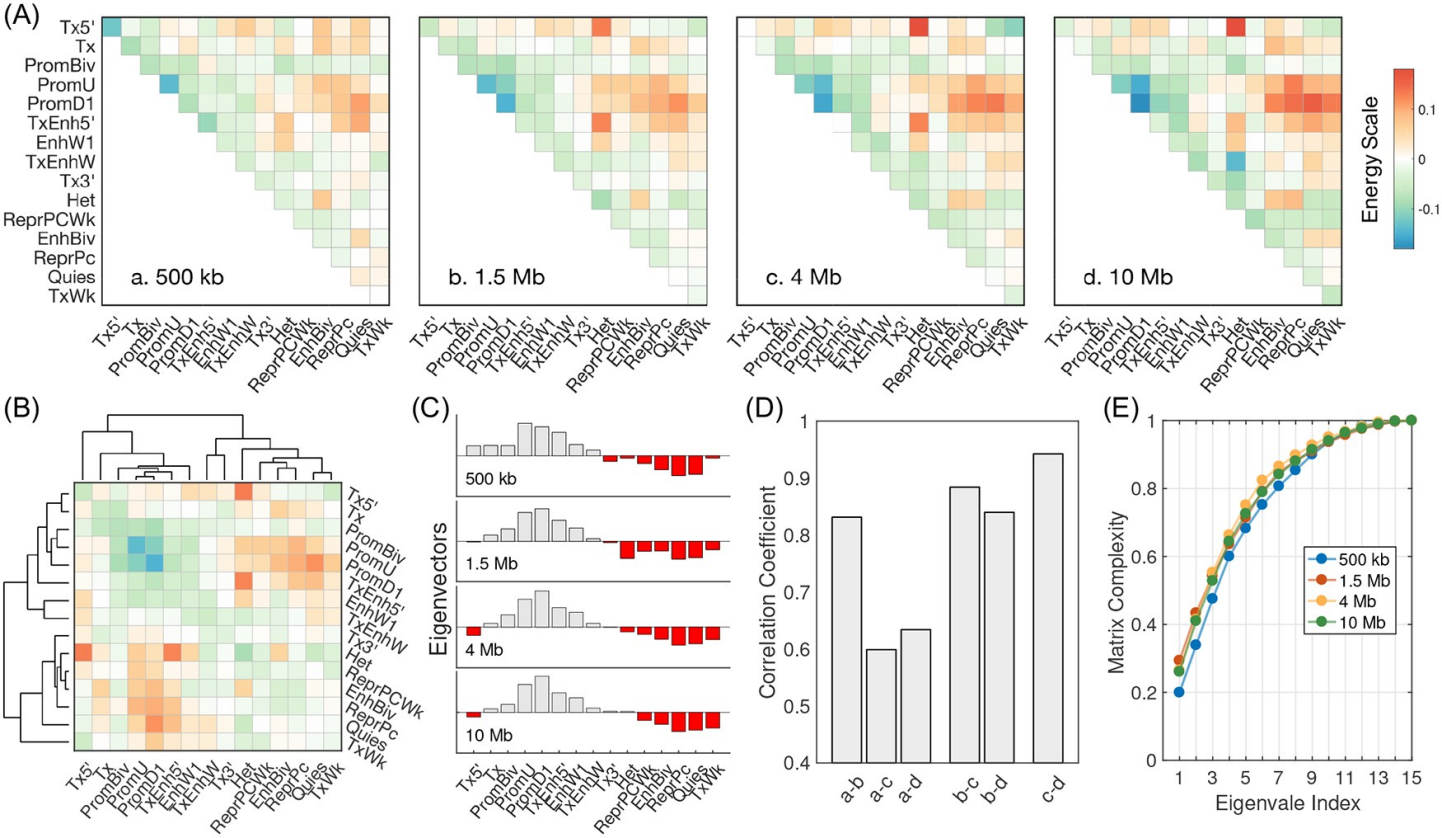
Dependence of chromatin state interaction energies on genomic separation. (A) Heat maps for the interaction matrices at various genomic separations, with blue and red corresponding to attractive and repulsive interactions respectively. We subtracted out the mean of the interaction energies in order to shift different plots to the same scale. (B) Dendrogram calculated using the interaction energy matrix at 1.5 Mb to highlight the hierarchical clustering of chromatin states. The coloring scheme is the same as in part (A). (C) The eigenvectors corresponding to the largest eigenvalues of the four interaction matrices, with grey and red indicating positive and negative values respectively. (D) Pearson correlation coefficients between interaction matrices at different scales. (E) The complexity measure for different interaction matrices as a function of the index for top eigenvalues. See text for the definition of the complexity measure.

Fig 7A presents the derived contact energies among chromatin states *U*_CS_(***r***) at various genomic separations (500kb, 1.5 Mb, 4 Mb and 10 Mb from left to right), with blue and red for attractive and repulsive interactions respectively. A notable feature for all four length scales is the clear partition of chromatin states into at least two groups that correspond to well-known active and repressive chromatins respectively. For example, attractive interactions are observed among the top half chromatin states that include promoters (PromD1, PromU), enhancers (TxEnh5, Enhw1) and gene body (Tx), and for the bottom half that includes inactive chromatin (Quies), polycomb repressed domain (ReprPC) and heterochromatin (Het). The unfavorable interactions among active and repressive chromatins will drive their phase separation shown in Fig 2D and Figure L in S1 Supporting Information. Partitioning of chromatin states into active and inactive groups is also evident from the dendrogram shown in Fig 7B, and the eigenvectors for the largest in magnitude eigenvalue of the interaction matrices shown in Fig 7C.

Despite their overall similarities, the interaction energies at various genomic separations differ from each other. To quantify their differences, we determined the pairwise Pearson correlation coefficients between the interaction matrices. As shown in Fig 7C, the interactions that are responsible for TAD formation (~ 1 Mb) indeed differ significantly from those that lead to chromatin compartmentalization (~ 10 Mb), as evidenced by the low correlation among them. Strikingly, the correlation coefficient between interaction matrices at 4 Mb and 10 Mb exceeds 0.9, indicating the convergence of chromatin interactions at large genomic separation.

We further compared the complexity of the interaction matrices by calculating the ratio of the first *n* eigenvalues over the sum of all eigenvalues. Fig 7D plots this complexity measure as a function of *n*, and absolute values of the eigenvalues were used to calculate the measure. For all three matrices with genomic separation larger than 1 Mb, we find the top first six eigenvectors can explain a large fraction of their complexity (over 80%). This observation is consistent with the success of our previous effort in modeling chromatin organization with six compartment types [37]. However, more eigenvectors are needed, especially for short range in sequence interactions, to capture the full matrix complexity. These results together highlight the presence of distinct mechanisms that fold the chromatin at various genomic separations, and argues the importance of using sequence length dependent contact energies.

## Discussion

We introduced a novel computational model for studying 3D genome organization by integrating bioinformatics analysis with polymer modeling. This integration brings together the best of both worlds and results in a powerful predictive tool. Similar to bioinformatics approaches, our model succeeds in identifying cell-type specific interactions between regulatory elements. As in polymer modeling, the availability of 3D chromosome conformations makes it possible to characterize contacts between any genomic segments and construct the whole contact map, to study global properties of the genome organization that involve many-body interactions, and to explore the physical mechanism and driving force of genome folding.

This predictive model presents a significant improvement from our previous effort in simulating chromatin structures [37] by switching the input from compartment types to chromatin states. In particular, unlike compartment types that are results from clustering Hi-C contact matrices [7], chromatin states are defined as combinational patterns of histone modification profiles. Uncoupling the input from Hi-C data is critical to ensure that the model is genuinely predictive. Furthermore, chromatin states allow us to model chromatin structures at a much higher resolution (5kb) to provide a detailed structural characterization of chromatin loops and TADs, and to resolve long-range specific contacts between promoters and enhancers. On the other hand, chromatin models based on compartment types are inherently limited to 50kb [37,39], a resolution at which compartment types can be robustly derived from Hi-C data [7]. Finally, as shown in Fig 7, the novel sequence-separation dependent contact potential developed here enables a rigorous assessment of the number of “types” needed for modeling chromatin structures, and suggests that the six compartment types are insufficient for an accurate description of TAD formation. Since the data required to define chromatin states are available for hundreds of cell types via the epigenome roadmap project [89], we anticipate a straightforward application of the model developed here to characterize the differences of chromatin structures across cell types and to understand the role of 3D genome organization in cell differentiation and cell fate establishment.

Histone modifications have long been recognized as crucial for the genome’s function [90]. The “histone code” hypothesis was proposed to rationalize the presence of numerous types of histone marks and the importance of their combinatorial roles [91]. However, a mechanistic understanding of the relationship between these chemical modifications and the functional outcome remains lacking [92]. The success of the computational model introduced here in predicting chromatin structures argues for the importance of histone modifications in organizing the genome. It is tantalizing to hypothesize that the histone code can be understood from a structural perspective. Epigenome engineering experiments that perturb histone modifications at specific genomic locations will be helpful to elucidate further whether the relationship between 1D histone modifications and 3D genome organization is causal.

## Methods

### Energy function of the chromatin model

The energy function of the chromosome model, which can be rigorously derived following the maximum entropy principle [64,65], adopts the following form

$$U_{Chrom}(r)=U(r)+\sum_{I,J}\sum_{i\in I}\sum_{j\in J}\alpha^{IJ}(\left|j-i\right|)f(r_{ij})+\;\sum_{K,L}\sum_{K\leq k<l\leq L}\left[\alpha_{Ch,Ch}+\alpha_{C,Ch}+\alpha_{C,C})f(r_{kl}\right].$$

*U*(***r***) defines the generic topology of the chromosome as a confined polymer with excluded volume effect. The second term incorporates the sequence length dependent contact energies *α*^*IJ*^ (|*j* − *i*|) between pairs of loci *i*, *j* characterized with chromatin states *I*, *J* respectively. As discussed in the main text, the dependence of contact energies on sequence length separation is crucial to reproduce the hierarchical genome organization, and to detect independent mechanisms of chromatin folding at different length scales. *f*(*r*_*ij*_) measures the contact probability between a pair of loci *i* and *j* separated by a distance *r*_*ij*_, and is defined as follows

f(r)={12[1+tanh(σ(rc-r))],ifr≤rc12(rcr)4,forr>rc#

Where *r*_*c*_ = 1.76 and *σ* = 3.72. As shown in Figure O in S1 Supporting Information, compared to a simple hyperbolic tangent function used in previous studies [64,65], the new expression decays to zero for large distances *r* at a slower rate. This new form is motivated by the power law relationship between spatial distances and Hi-C contact probabilities observed in Ref. [13].

Finally, the last term, inspired by the recently proposed extrusion model [29–31], is included to model the formation of chromatin loops. In particular, the genomic segment enclosed by a pair of convergent CTCF binding sites experiences a condensing potential due to the binding of cohesin molecules. We limit this potential to convergent CTCF pairs that are separated by no more than 4 CTCF binding sites with 5’– 3’ orientation or 4 CTCF binding sites with 3’– 5’ orientation to mimic the finite processivity of cohesin molecules [30]. For generality, three different potentials are used for CTCF-CTCF interaction (*α*_C,C_), CTCF-chromatin interaction (*α*_C,Ch_) and chromatin-chromatin interaction (*α*_Ch,Ch_).

The explicit mathematical expression for *U*_Chrom_(***r***) is provided in the SI. It contains a total of 1883 parameters. This seemingly large number is a result of our use of chromatin states and the dependence of their interaction energies, *α*^*IJ*^ (|*j* − *i*|), on genomic separation. Both of these two features are innovations of our model to predict specific contacts between enhancers and promoters, and to capture the different biological mechanisms for TAD formation and chromosome compartmentalization. We emphasize that since a specific experimental constraint can be defined for each one of these parameters, their values can be derived robustly and efficiently using the iterative maximum entropy algorithm introduced by Zhang and Wolynes [64]. As proven before, the value of these parameters in principle is unique [76]. Numerical values of the parameters are provided in the Extended Data Sheet.

After a careful analysis of the interaction energies shown in Fig 7, however, we believe that the number of parameters could potentially be significantly reduced without sacrificing the model accuracy. In particular, the number of chromatin states used here is probably “too many” since the complexity of the interaction energy matrices can be well explained with the top 10 eigenvectors. Furthermore, the interaction energies also converge at larger genomic separation, making its dependence on |*j* − *i*| unnecessary. These insights will prove useful for future chromatin modeling efforts.

### Simulation details

We carried out constant temperature simulations to predict chromatin structures consistent with the energy function *U*_Chrom_(***r***) using the molecular dynamics software package LAMMPS [93]. For each contact map presented in the manuscript, a total of eight independent 40-million-timestep long simulations were performed to ensure sufficient statistics. On an Intel Xeon E5-2690 v4 2.6GHz node with 14 cores, each one of such simulations takes approximately 30 hours to finish. More details on the simulation are provided in the supporting information.

To enable a quantitative comparison between simulated chromatin structures with microscopy imaging data, we estimate a 5kb long genomic segment with a width of 30 nm and a length of 60 nm based on a high-resolution chromatin structure characterized by cryogenic electron microscopy (Cryo-EM) technique [94].

### Hi-C data analysis

Experimental contact maps at 5kb resolution from Ref. were downloaded using the Gene Expression Omnibus (GEO) accession number GSE63525 (see Extended Data Sheet). We used the combined contact matrices constructed from all read pairs that map to the genome with a MAPQ> = 30. The raw matrices were then normalized with the KR method using the normalization vector provided in the same dataset. To convert the contact matrices into probabilities, we further divided each matrix element with the diagonal value *C*_*ii*_ = 1035 obtained from averaging over all chromosomes. With this probability conversion, all the genomic segments that are within in 5kb along the sequence will on average have a contact probability of 1. Since in the computational model, a 5kb segment has a diameter of *σ* = 30 nm, this probability conversion is equivalent of specifying the contact probability as 1 for genomic loci that are within a spatial distance of 30 nm. Such a probability definition is indeed consistent with the contact function *f*(*r*) defined in Eq. [3] and plotted in Figure O in S1 Supporting Information.

### Chromatin states from epigenomics data

A key input of the computational model is the sequence of chromatin states that captures the variation of epigenetic modifications along the genome sequence. Following Ref. [67], we defined chromatin states as the set of unique combinatorial patterns of histone marks. Using a multivariate hidden Markov model that maximizes the posterior probability of assigning a hidden state to each genomic segment given the sequence of observed histone modifications [95], we derived 15 chromatin states from genome-wide profiles of 12 key histone marks collected from six cell types that include GM12878, K562, HeLa, H1hesc, Huvec and Hepg2. A single set of chromatin states is crucial to ensure the transferability of the parameterized force field across cell types. The dataset used for chromatin state inference is listed in the Extended Data Sheet. Detailed histone modification patterns for these chromatin states are shown in Fig 1C. With the set of chromatin states specified, every five-kilo-base long segment can then be assigned to a chromatin state based on its histone modification profiles, and a sequence of chromatin states for the entire chromatin segment can be defined as the simulation input.

### Genomic locations and orientations of CTCF binding sites from ChIP-Seq data

To capture the formation of chromatin loops, we compiled a list of CTCF-binding sites along the chromatin of interest using cell-type specific ChIP-Seq data.

Starting from the peak profile downloaded from ENCODE (see Extended data sheet), we identified the center of binding for each peak of both CTCF and cohesin subunit Rad21. As both CTCF and cohesin molecules are found at the boundaries of most chromatin loops, we selected loop forming CTCF binding sites as those that have at least one Rad21 molecule located within 50bp of their genomic locations.

We then determined the orientation of each CTCF-binding site as follows. We first attempted to align the binding sites to the set of CTCF motifs compiled in Refs. [7] and [96] (see Extended data sheet). If the alignment succeeds and a motif is found within 100bp of the binding site, the orientation of the binding site was then assigned based on the DNA sequence of that motif. If no motif can be aligned, the orientation of the CTCF-binding site is determined using the genomic location of its binding center relative to that of the nearest binding center of Rad21. For example, we assign the orientation as 5’– 3’ if the nearest Rad21 binding center is in the downstream of the CTCF binding site; otherwise, the orientation is assigned as 3’– 5’.

The above procedure will result in a list of oriented CTCF sites at single base resolution. From this list, we defined a 5kb-long bead in the computational model as a CTCF site if there is at least one CTCF binding site falls into the genomic region enclosed by that bead. If all the CTCF sites within the 5kb region have the 5’– 3’ orientation, then the bead is assigned with the 5’– 3’ orientation; similarly, if all the CTCF sites within the 5kb region have the 3’– 5’ orientation, then the bead is assigned with the 3’– 5’ orientation. If CTCF sites with both orientations are present, then the bead is assigned with dual orientation as well.

### Diffusion map analysis

For molecular systems that exhibit a separation of timescales, it is often desirable to approximate their dynamics at long time limit with a handful of slow variables. The time evolution of these slow variables should be Markovian and independent of the fine details of the high dimensional system to capture the dynamical behavior of the system on a coarsened timescale. Mathematically it has been proven that an optimal choice of these slow variables is the first few eigenfunctions of the backward Fokker–Planck diffusion operator [81]. Diffusion map is a data-driven approach that approximates these eigenfunctions and therefore the slow variables by defining a random walk process on the simulation data [97].

In particular, for *N* chromatin configurations selected from the simulated structural ensemble, we first constructed a transition probability matrix *K* for the random walk by defining its elements as

$$K_{ij}=\text{exp}\left(-\frac{d_{ij}}{2\epsilon_{i}\epsilon_{j}}\right).$$

The eigenfunctions of the above transition matrix can be shown to converge to that of the Fokker–Planck operator in large *N* limit. The distance between two configurations *d*_*ij*_ was calculated as the mean difference of their corresponding contact probability maps. We followed the algorithm proposed in Ref. [82] to normalize the matrix and to estimate *ϵ*_*i*_. From the normalized transition matrix, we then determined its eigenfunctions and used the top two with the smallest non-zero eigenvalues as the reaction coordinates shown in Fig 6 (see Figure N in S1 Supporting Information for eigenvalues).

## Supporting information

S1 Supporting Information Additional model details and validations. (PDF)

S1 Table Extended data sheets. Source for experimental data used in the study.
(XLSX)
