# Supplementary material for: Predicting three-dimensional genome organization with chromatin states
Source: PLoS Comput Biol. 2019 Jun 10;15(6):e1007024. doi: 10.1371/journal.pcbi.1007024 (PMC6586364; doi:10.1371/journal.pcbi.1007024)
Supplement: S1 Supporting Information — (PDF) [file pcbi.1007024.s001.pdf]

Supporting Information for "Predicting three-dimensional genome organization with chromatin states"

Authors: Yifeng Qi, Bin Zhang*

Affiliations:

Departments of Chemistry, Massachusetts Institute of Technology, 77 Massachusetts Ave, Cambridge, MA 02139-4307

*Correspondence to: binz@mit.edu.

Energy function of the chromatin model. As mentioned in the main text, we model the chromatin as beads on a string. The potential energy for a given chromatin configuration $\boldsymbol{r}$ consists of three components

$$U_{\text{Chrom}}(\boldsymbol{r}) = U(\boldsymbol{r}) + U_{\text{CS}}(\boldsymbol{r}) + U_{\text{CTCF}}(\boldsymbol{r}) \quad [1]$$

$U(\boldsymbol{r})$ is the energy function for a confined homopolymer that consists of the following four terms, U_{bond}, U_{angle}, U_{sc} and U_{c}.

U_{bond} is the bonding potential between neighboring beads and is defined as

$$U_{\text{bond}} = K_2(r - r_o)^2 + K_3(r - r_o)^3 + K_4(r - r_o)^4,$$

where $K_2 = K_3 = K_4 = 20\frac{\epsilon}{\sigma^2}$ and $r_o = 2.0\ \sigma$. $\sigma = 30$ nm is the diameter of the bead. We choose the distance between neighboring beads as 60 nm to arrive at a nucleosome line density $0.43\ nm^{-1}$ that is consistent with the recent chromatin fiber structure resolved at 11 Å with cryo-EM [1]. $\epsilon = k_B T$ defines the energy scale of the model.

U_{angle} is an angular potential that defines the persistence length of the polymer. It is applied to all connected three consecutive monomers in the following form

$$U_{\text{angle}} = K_a[1 - \cos(\theta - \pi)],$$

where $K_a = 2\ \epsilon$.

U_{sc} is a soft-core potential applied to all the non-bonded pairs to enforce the excluded volume effect among genomic loci, and is defined as

$$U_{\text{sc}} = \begin{cases} 0.5E_{\text{cut}}\left(1 + \tanh\left[\dfrac{2U_{\text{LJ}}(r)}{E_{\text{cut}}} - 1\right]\right), & r \le r_{\text{cut}} \\ U_{\text{LJ}}(r), & r_{\text{cut}} \le r \le \sigma 2^{1/6}, \\ 0, & r > \sigma 2^{1/6}. \end{cases}$$

The above expression corresponds to the Lennard-Jones potential $U_{\mathrm{LJ}}(r) = 4\epsilon\left[\left(\frac{\sigma}{r}\right)^{12} - \left(\frac{\sigma}{r}\right)^{6}\right] + \epsilon$ capped off at a finite volume within a repulsive core to allow for chain crossing at finite energetic cost. r_{cut} is chosen as the distance at which $U_{\mathrm{LJ}}(r) \equiv 0.5\ E_{\mathrm{cut}}$, and $E_{\mathrm{cut}} = 4\epsilon$.

U_{c} is introduced to mimic the confinement effect that chromosomes experience inside the cell due to their interaction with the nuclear envelope. It is defined as a spherical boundary whose radius r_{confine} is chosen to maintain a given base pair density ρ. Each chromatin bead interacts with its nearest point on the boundary through a hard-core potential U_{c}.

$$U_{\mathrm{c}} = \begin{cases} 4\epsilon\left[\left(\frac{\sigma}{r}\right)^{12} - \left(\frac{\sigma}{r}\right)^{6}\right] + \epsilon, r \leq \sigma 2^{1/6} \\ 0, r > \sigma 2^{1/6} \end{cases}$$

We initialize all the computer simulations with a chromatin configuration in which all beads are inside the boundary. Because of the hard-core potential, these beads will then remain inside the confinement during the entire simulation. The radius of the confinement is chosen to approximate the base pair density found in the human nucleus that encloses ~ 6 billion base pairs in a volume of $10\mu m$ in diameter. We therefore estimate the density as $\rho = \frac{6.176\times 10^{9}\mathrm{bp}}{\left(\frac{4}{3}\pi(5\mu\mathrm{m})^{3}\right)} = 1.1795 \times 10^{7}\frac{bp}{\mu m^{3}}$, and set confinement size to be $0.79686\ \mu m = 26.562\ \sigma$ for the simulated chromosome segment of 25 Mb in length.

The two additional terms in Eq. [1] are introduced to model the genome organization starting from one-dimensional sequence features of the chromatin. In particular, $U_{\mathrm{CS}}(\boldsymbol{r})$ quantifies chromatin state specific interaction energies between pairs of loci, and is defined as

$$U_{\mathrm{CS}}(\boldsymbol{r}) = \sum_{IJ}\sum_{i\in I}\sum_{j\in J}\alpha^{IJ}(|j-i|)\, f(r_{ij}), \qquad [2]$$

where I and J indexes different chromatin states, and i and j runs over different genomic segments. This chromatin state-based potential is crucial for the formation of topologically associating domains (TADs) and the compartmentalization of chromatin domains.

$f(r_{ij})$ in Eq. [2] determines the probability to record a contact between a pair of genomic segments i and j separated by a distance r_{ij}, and is defined as follows

$$f(r) = \begin{cases} \frac{1}{2}\left[1 + \tanh\left(\sigma(r_c - r)\right)\right], \mathrm{if}\ r \leq r_{\mathrm{c}} \\ \frac{1}{2}\left(\frac{r_c}{r}\right)^{4}, \ \mathrm{for}\ r > r_{\mathrm{c}} \end{cases} \qquad [3]$$

where $r_c = 1.76$ and $\sigma = 3.72$. As shown in Figure O, compared to a simple hyperbolic tangent function used in previous studies [2,3], the new expression decays to zero for large distances r at a slower rate. This new form is motivated by the power law relationship between spatial distances and Hi-C contact probabilities observed in Ref. [4].

The prefactor $\alpha^{IJ}(|j-i|)$ in Eq. [2] measures the energetic cost for forming a contact between a pair of genomic loci i and j. It depends explicitly on the chromatin states I and J for the two loci and also the genomic separation $|j-i|$ between them. As mentioned in the main text, the dependence of these contact energies on genomic separation is crucial to distinguish different mechanisms that lead to the formation of TADs at the intermediate scale, and the compartmentalization of chromatin domains separated far apart in sequence.

In its most general form, $\alpha^{IJ}(|j-i|)$ would require too many parameters to be parameterized robustly. For example, with 15 chromatin states and a chromosome of 4000 beads (20 Mb in sequence length), the parameter number would be $\frac{15(15+1)}{2} \times 4000 = 480000$. We therefore introduce the following strategy to reduce the number of parameters. First, we separate $\alpha^{IJ}(|j-i|)$ into two terms that include a mean field ideal potential α_{ideal} and a sequence specific potential α_{seq}

$$\alpha^{IJ}(|j-i|) = \alpha_{\text{ideal}}(|j-i|) + \alpha_{\text{seq}}^{IJ}(|j-i|). \qquad [4]$$

We then approximate the two potential terms with different approaches. For the ideal potential, instead of defining its value at every sequence separation with a 5 kb increment, we use a coarsened grid with a spacing of 50kb and approximate all the potential in the same grid with a single value. For example,

$$\alpha_{\text{ideal}}(|j-i|) \equiv \alpha_{\text{ideal}}^{\text{coarse}}(10(s-1)+1)$$
$$\text{for } |j-i| \in [10(s-1)+1, 10s] \text{ and } s \in \left[1, \frac{N}{10}\right],$$

where N is the total number of polymer beads. On top of the coarse grid, we add 40 more parameters to refine the ideal potential at a 5kb resolution for sequence separations $|j-i|$ that are less than 200 kb. The final expression for $\alpha_{\text{ideal}}(|j-i|)$ with $|j-i| \in [10(s-1)+1, 10s]$ can be summarized as

$$\alpha_{\text{ideal}}(|j-i|) = \begin{cases} \alpha_{\text{ideal}}^{\text{coarse}}(10(s-1)+1) + \alpha_{\text{ideal}}^{\text{fine}}(|j-i|), & s \leq 40 \\ \alpha_{\text{ideal}}^{\text{coarse}}(10(s-1)+1), & s > 40. \end{cases}$$

A total of 440 parameters is thus used to define $\alpha_{\text{ideal}}(|j-i|)$.

For each pair of the chromatin states I and J, we approximate the sequence specific potential with four piece-wise terms,

$$\alpha_{\text{seq}}^{IJ}(|j-i|) = \begin{cases} a_1^{IJ} + b_1^{IJ}\ln(|j-i|) + c_1^{IJ}|j-i|^{0.25}, & \text{for } |j-i| \le 1\text{Mb} \\ a_2^{IJ} + b_2^{IJ}\ln(|j-i|) + c_2^{IJ}|j-i|^{0.25}, & \text{for } 1\text{Mb} < |j-i| \le 2\text{Mb} \\ a_3^{IJ} + b_3^{IJ}\ln(|j-i|) + c_3^{IJ}|j-i|^{0.25}, & \text{for } 2\text{Mb} < |j-i| \le 5\text{Mb} \\ a_4^{IJ} + b_4^{IJ}\ln(|j-i|) + c_4^{IJ}|j-i|^{0.25}, & \text{for } 5\text{Mb} < |j-i| \le 20\text{Mb} \end{cases} \quad [5]$$

The functional form $a + b\ln(|j-i|) + c|j-i|^{0.25}$ is chosen to provide a good fit for the power-law decay $|j-i|^{\alpha}$ for most $\alpha \in [0,1]$ by varying the coefficients a, b and c. Power-law decay is a reasonable approximation to the dependence of contact energies on sequence length separation. The four different sets of parameters make it possible to capture a possible change in α over the entire range of genomic separation studied. With the above definition, a total number of $\frac{15(15+1)}{2} \times 3 \times 4 = 1440$ parameters is needed for the sequence-specific term.

The last term in Eq. [1], $U_{\text{CTCF}}(\boldsymbol{r})$, is included to model the interaction between pairs of genomics loci k and l due to the formation of chromatin loops anchored by pairs of CCCTC-binding factor (CTCF), and is defined as

$$U_{\text{CTCF}}(\boldsymbol{r}) = \sum_{K,L}\sum_{K\le k<l\le L}\left[\alpha_1(1-\delta_{k,K})(1-\delta_{l,L}) + \alpha_2(\delta_{k,K}+\delta_{l,L})(1-\delta_{k,K}\delta_{l,L}) + \alpha_3\delta_{k,K}\delta_{l,L}\right]f(r_{kl}), \quad [6]$$

where δ is Kronecker delta function and K (L) indexes over CTCF binding sites with 5' – 3'(3' – 5') orientation. The particular functional form for $U_{\text{CTCF}}(\boldsymbol{r})$ is motivated by the extrusion model [5–7]. For example, since binding of cohesin molecules to two strands of chromatin will bring them into spatial proximity, the effect of this binding can be approximated as an effective attraction between genomic loci. Since cohesin can extrude linearly along the chromosome within the region bound by a pair of convergent CTCF-binding sites, this effective interaction will also be limited to genomic loci enclosed by CTCF-binding sites (1st term). We used two additional parameters to model the interaction between CTCF-binding sites and the enclosed chromatin (2nd term), and interaction between CTCF-binding sites (3rd term). Since cohesin molecules have longer residence time near the CTCF molecules, the effective interaction between CTCF-binding sites and the chromatin may be stronger compared to chromatin-chromatin interaction, and therefore requires a separate potential term. We note that $U_{\text{CTCF}}(\boldsymbol{r})$ is only applied for convergent CTCF pairs that are separated by no more than 4 CTCF binding sites with 5' – 3' orientation or 4 CTCF binding sites with 3' – 5' orientation to mimic the finite processivity of cohesin molecules.

Parameter optimization. Though the chromatin energy function $U_{\text{Chrom}}(\boldsymbol{r})$ in Eq. [1] has clear physically meanings and is biologically justified, its expression can also be derived following the maximum entropy framework proposed in Refs. [2,3]. In particular, $U_{\text{Chrom}}(\boldsymbol{r})$ can be shown as the least biased functional form to reproduce the following set of constraints that include the average contact probabilities between a generic pair of genomic loci at various sequence separations, average contact probabilities between pairs of chromatin states I and J at various sequence

separations, and average contact probabilities between pairs of CTCF binding sites K and L and between chromatin segments enclosed by convergent CTCF pairs

$$
\begin{aligned}
\textstyle\sum_{i,j}\langle f(r_{ij})\delta_{|j-i|,s}\rangle &= \textstyle\sum_{i,j} f_{ij}^{\text{exp}}\,\delta_{|j-i|,s} \\
\textstyle\sum_{i\in I}\sum_{j\in J}\langle f(r_{ij})\delta_{|j-i|,s}\rangle &= \textstyle\sum_{i\in I}\sum_{j\in J} f_{ij}^{\text{exp}}\delta_{|j-i|,s} \\
\textstyle\sum_{K,L}\langle f(r_{KL})\rangle &= \textstyle\sum_{K,L} f_{KL}^{\text{exp}} \\
\textstyle\sum_{K}\sum_{K<l<L}\langle f(r_{Kl})\rangle + \sum_{L}\sum_{K<k<L}\langle f(r_{kL})\rangle & \\
&= \textstyle\sum_{K}\sum_{K<l<L} f_{Kl}^{\text{exp}} + \sum_{L}\sum_{K<k<L} f_{kL}^{\text{exp}} \\
\textstyle\sum_{K,L}\sum_{K<k<l<L}\langle f(r_{kl})\rangle &= \textstyle\sum_{K,L}\sum_{K<k<l<L} f_{kl}^{\text{exp}}
\end{aligned} \qquad [7]
$$

for $s = 1, \dots, N,\ \ I, J = 1 \dots N_{\text{cs}},\ \ K = 1, \cdots, N_{\text{CTCF}}^{53}$, and $L = 1, \cdots, N_{\text{CTCF}}^{35}$.

In the above equation, δ is the Kronecker delta function, f_{ij}^{exp} is the contact probability between the pair of genomic segments i and j, N is the number of polymer beads, N_{cs} is the number of chromatin states, and N_{CTCF}^{53} N_{CTCF}^{35} are the number of CTCF binding sites oriented in the $5' - 3'$ and $3' - 5'$ directions respectively. The angular brackets $\langle\cdot\rangle$ represent ensemble averages over the Boltzmann distribution $e^{-\beta U_{\text{chrom}}(\boldsymbol{r})}$. Again, the summation over K, L in the 3rd, 4th and 5th equation is only applied for convergent CTCF pairs that are separated by no more than 4 CTCF binding sites with 5' – 3' orientation or 4 CTCF binding sites with 3' – 5' orientation.

The first constraint in Eq. [7] will give rise to the ideal potential $\alpha_{\text{ideal}}(|j-i|)$ introduced in Eq. [4]. As mentioned above, $\alpha_{\text{ideal}}(|j-i|)$ is defined on a coarsened grid with a spacing of 50kb to reduce the number of parameters. Correspondingly, the constraints can be modified for this coarsened definition as

$$
\sum_{i,j}\langle f(r_{ij})\Theta_{|j-i|,s}\rangle = \sum_{i,j} f_{ij}^{\text{exp}}\,\Theta_{|j-i|,s}, \qquad \text{for } s = 1, \dots, \frac{N}{10}, \qquad [8]
$$

where $\Theta_{|j-i|,s} = H\big(|j-i| - 10(s-1)\big) \times H(10s - |j-i|)$ and only has non-zero values in the region $|j-i| \in [10(s-1)+1, 10s]$. $H(x)$ is the Heaviside step function.

Similarly, we can adjust the second constraint in Eq. [7] for the approximate form of $\alpha_{\text{seq}}^{IJ}(|j-i|)$ defined in Eq. [5]. In particular, we can define the following constraints separately for each one of the four sequence ranges

$$
\begin{aligned}
\sum_{i\in I}\sum_{j\in J}\langle f(r_{ij})\delta_{|j-i|,s}\rangle &= \sum_{i\in I}\sum_{j\in J} f_{ij}^{\text{exp}}\delta_{|j-i|,s} \\
\sum_{i\in I}\sum_{j\in J}\langle f(r_{ij})\delta_{|j-i|,s}\ln|j-i|\rangle &= \sum_{i\in I}\sum_{j\in J} f_{ij}^{\text{exp}}\delta_{|j-i|,s}\ln|j-i| \\
\sum_{i\in I}\sum_{j\in J}\langle f(r_{ij})\delta_{|j-i|,s}|j-i|^{0.25}\rangle &= \sum_{i\in I}\sum_{j\in J} f_{ij}^{\text{exp}}\delta_{|j-i|,s}|j-i|^{0.25}
\end{aligned} \qquad [9]
$$

The maximum entropy principle also suggests a simple optimization algorithm to derive the parameters in the potential. The strengths of contact energies, $\boldsymbol{\alpha}$, are determined by minimizing the objective function defined as:

$$\begin{aligned}\Gamma(\boldsymbol{\alpha}) = \ln\left(\frac{Z(\boldsymbol{\alpha})}{Z_0}\right) \\ + \beta\Bigg(\sum_s \alpha_{\text{ideal}}(s)\sum_{i,j}\delta_{|j-i|,s} f_{ij}^{\exp} + \sum_{I,J}\sum_s \alpha_{\text{residual}}^{IJ}(s)\sum_{i\in I}\sum_{j\in J}\delta_{|j-i|,s} f_{ij}^{\exp} \\ + \sum_{K,L}\sum_{K\le k<l\le L}\left[\alpha_1(1-\delta_{k,K})(1-\delta_{l,L}) + \alpha_2(\delta_{k,K}+\delta_{l,L})(1-\delta_{k,K}\delta_{l,L})\right. \\ \left. + \alpha_3\delta_{k,K}\delta_{l,L}\right] f_{kl}^{\exp}\Bigg)\end{aligned} \quad [10]$$

where

$$Z(\boldsymbol{\alpha}) = \int d\boldsymbol{r}\, e^{-\beta U_{\text{Chrom}}(\boldsymbol{r})}$$
$$Z_o = \int d\boldsymbol{r}\, e^{-\beta U(\boldsymbol{r})}.$$

are the partition functions for $U_{\text{Chrom}}(\boldsymbol{r})$ and $U(\boldsymbol{r})$. For simplicity, $\Gamma(\boldsymbol{\alpha})$ is expressed in terms of the explicit constraints defined in Eq. [7]. Generalizing to the coarsened constraints defined in Eqs. [8] and [9] is straightforward. The objective function $\Gamma(\boldsymbol{\alpha})$ is a measurement of how much information theoretic entropy of the system is lost because of being constrained to the experimental input data. By maximizing $\Gamma(\boldsymbol{\alpha})$, the parameters in the potential $U_{\text{Chrom}}(\boldsymbol{r})$ can be found when $\frac{\partial\Gamma}{\partial\alpha} \equiv 0$. The same iterative algorithm outlined in the *Section: Inverse Statistical Mechanics and the Maximum Entropy Ensemble* of the supporting information from Ref. [2] was used for parameter optimization. As mentioned in the main text, we used Hi-C experiments for segments of chromosomes 1, 10, 19, and 21 from GM12878 cells to determine the experimental constraints $f^{\exp}(r_{ij})$.

Molecular dynamics simulation details. All simulations were carried out using the molecular dynamics package LAMMPS [8] with reduced units $\sigma = 30\ nm$ and $\epsilon = k_B T$. Simulations were maintained at a constant temperature $T = 1.0$ via Langevin dynamics with a damping coefficient $\gamma = 0.5\tau$ and a time step of $dt = 0.012\tau$, where τ is the time unit.

Due to computational costs, we only simulated continuous genomic regions that are of 25Mb in length instead of whole chromosomes. These regions were mostly chosen from the q arms to avoid centromere regions that lack Hi-C data, and their genomic positions are provided in the Extended Data Sheet.

As aforementioned, the chromatin is confined in a spherical boundary during the simulation to mimic its interaction with the nuclear envelope. Such a confinement, however, also introduce boundary effects to the two end regions of the chromatin. Unlike the central regions, these two ends will experience more interaction with the boundary. To alleviate this boundary effect, we

simulated a chromosome of 25Mb in length, but only used the middle 20Mb segment for analysis, and discarded the two end regions 0-2Mb and 22-25Mb.

To generate the initial configuration of these simulations, we first built a random polymer structure inside a spherical confinement. We then performed equilibration simulation using only the homopolymer potential, $U(r)$, to relax both the topology and energy of the polymer structure. The last configuration from this equilibration simulation is then used for our chromosome simulations.

To calculate the ensemble averages during the parameter optimization step, we carried out eight independent 20-million-time-step-long simulations for each one of the four chromosomes. The starting configurations for these simulations were chosen as the end configurations from the last iteration. All simulations in the first iteration started from a random polymer configuration confined in the spherical boundary.

For all the predictions, we carried out eight independent simulations, each of which lasted 40 million-time steps.

Contact enhancement metric for chromatin loops. Chromatin loops are identified as pairs of genomic loci whose contact probabilities measured in Hi-C experiments are significantly higher than the local background signal. Based on this definition, we use the ratio of the average contact probability of the peak region over that of the local background region as a measure of loop prediction quality. For a loop to stand out from the background, this ratio should be significantly higher than that from a randomly selected pair of loci. An illustration of the definition of peak and local background region is provided in Figure F1A. We define the peak and background regions as the areas enclosed by green and black squares respectively. The exact coordinates used to specify these regions relative to the genomic positions of the two loop anchors $(0, 0)$ are marked on the right panel of Figure F1A. We define the average contact probabilities over all the pixels in the peak regions as P_{p}, and the two background regions as P_{b}. The contact enhancement of a chromatin loop is then calculated as the ratio of the contact probabilities $\frac{P_{\mathrm{p}}}{P_{\mathrm{b}}}$.

As a comparison, we also calculated the contact enhancements for a list of randomly selected genomic pairs. To generate these random pairs, we first selected their starting genomic positions from the simulated chromatin region with equal probability. The genomic separation for these random pairs were then determined based on a Gamma distribution obtained from a numerical fit to the length distribution of chromatin loops found in Hi-C contact maps, $P(l) = \frac{1}{b^a \Gamma(a)} l^{a-1} e^{-\frac{x}{b}}$, with $a = 1.69$ and $b = 175.19$ (see Figure F1B).

De novo **detection of TADs and significant contacts.** we performed additional analysis using existing software to further validate the model's accuracy in predicting TADs and loops. In particular, we used two methods to examine the quality of the TAD boundaries predicted by simulated Hi-C contact maps at 50kb resolution. *First*, we used the TADbit developed by the Marti-Renom group [9] to directly identify TADs by optimizing the BIC-penalized likelihood calculated from Hi-C contacts for individual chromosomal segments. As shown in Figure I, the boundaries for TADs detected from simulated (top) and experimental Hi-C data (bottom) are in

good agreement with each other. As an additional measure, we calculated the insulation score introduced by Dekker and co-workers [10]. A sliding window of 500kb in size was used along the off diagonal to determine the average contact probability as a function of genomic distance. For the insulation score, TAD boundaries were identified from its local minima. The simulated and experimental insulation scores are again in good agreement.

To more quantitatively measure the agreement between simulation and experiment, we defined a metric termed as matching score. An experimental TAD is matched with a simulated one if their boundaries overlap. A deviation of 5 bins was allowed when determining the overlap between boundaries to take into account the effect of statistical noise that can result in uncertainties for boundary detection. The matching score is determined as the fraction of experimental TADs that is captured by simulation. The reverse matching score can be similarly defined as the percent of simulated TADs found in the experimental ones. As shown in Figure K1A, for most of the chromosomes, over 50% of the TADs are well described by simulation. Interestingly, using the boundaries identified from the insulation score, we found that over 70% of the boundaries are detected in our simulation (see Fig 3D and Figure K1B). This discrepancy highlights the challenge in robustly detecting TADs from noisy data. In fact, using a different algorithm, ArrowHead, developed by the Aiden group [11], we find that less than 40% of the TADs detected by arrowhead agree with the ones from TADbit, even though both were applied to the same experimental data (see Figure K1C). Given this challenge, we claim that TADs are well reproduced by our model.

We further used the software Fit-Hi-C developed by the Noble group [12] to perform loop calling over contact maps constructed at the 10kb resolution. We note that Fit-Hi-C detects significant contacts with high statistical confidence purely based on Hi-C data. Therefore, the contacts identified may differ from the typical chromatin loops that are frequently flanked by convergent CTCF motifs. As shown in Figure G, the top 1000 most significant contacts detected from simulation and experiment are in good agreement. In particular, as shown in Fig 3E and Figure K2A, over 40% of the experimental contacts can be precisely matched in the simulation. The matching score is defined similarly as that for TADs, we again allowed deviations within 5 bins (50kb) when determining the matches. Furthermore, if we relax the matching criterion for one of the two boundaries from 50kb to 400kb, the matching score increases to over 90% (Figure K2B). Together with Figure G, this quantitative analysis suggests that the simulation succeeds in identifying interacting domains; though many of the specific contacts within these domains are correctly predicted, a significant amount of them is not well described. Further improving the prediction accuracy of specific contacts within a domain would potentially require the inclusion of additional transcription factors, and would be an interesting future direction.

Clustering analysis of simulated chromosome structures. To investigate the spatial co-localization of different chromatin states, we performed the following clustering analysis using the algorithm introduced in Ref. [13]. Briefly, we identified the clusters as the set connected networks formed among genomic loci. Edges in these networks were defined between nearest neighbor loci as determined using Voronoi tessellation [14].

For simplicity, the clustering analysis was performed for five coarse chromatin types defined by grouping the 15 chromatin states introduced in Figure A1: Active 1 (Tx3, Tx, TxEnhW, Tx5),

Active 2 (TxEnh5, PromD1, PromU, EnhW1), Bivalent (PromBiv, EnhBiv, TxWk), Repressive (ReprPC, Het), and Inactive (ReprPCWk, Quies).

References:

1. Song F, Chen P, Sun D, Wang M, Dong L, Liang D, et al. Cryo-EM Study of the Chromatin Fiber Reveals a Double Helix Twisted by Tetranucleosomal Units. Science. 2014;344: 376 LP-380.
2. Zhang B, Wolynes PG. Topology, structures, and energy landscapes of human chromosomes. Proc Natl Acad Sci. 2015;112: 6062–6067.
3. Zhang B, Wolynes PG. Shape Transitions and Chiral Symmetry Breaking in the Energy Landscape of the Mitotic Chromosome. Phys Rev Lett. 2016;116: 248101.
4. Wang S, Su J-HH, Beliveau BJ, Bintu B, Moffitt JR, Wu CT, et al. Spatial organization of chromatin domains and compartments in single chromosomes. Science. 2016;353: 598–602.
5. Fudenberg G, Imakaev M, Lu C, Goloborodko A, Abdennur N, Mirny LALA. Formation of Chromosomal Domains by Loop Extrusion. Cell Rep. 2016;15: 2038–2049.
6. Goloborodko A, Marko JF, Mirny LA. Chromosome Compaction by Active Loop Extrusion. Biophys J. 2016;110: 2162–2168.
7. Sanborn AL, Rao SSP, Huang S-CC, Durand NC, Huntley MH, Jewett AI, et al. Chromatin extrusion explains key features of loop and domain formation in wild-type and engineered genomes. Proc Natl Acad Sci USA. 2015;112: E6456-6465.
8. Plimpton S, National LS. Fast Parallel Algorithms for Short–Range Molecular Dynamics. J Comput Phys. 1995;117: 1–42.
9. Serra F, Baù D, Goodstadt M, Castillo D, Filion GJ, Marti-Renom MA. Automatic analysis and 3D-modelling of Hi-C data using TADbit reveals structural features of the fly chromatin colors. PLoS Comput Biol. 2017;13: e1005665.
10. Crane E, Bian Q, McCord RP, Lajoie BR, Wheeler BS, Ralston EJ, et al. Condensin-driven remodelling of X chromosome topology during dosage compensation. Nature. 2015;523: 240.
11. Durand NCC, Shamim MSS, Machol I, Rao SSPSP, Huntley MHH, Lander ESS, et al. Juicer Provides a One-Click System for Analyzing Loop-Resolution Hi-C Experiments. Cell Syst. 2016;3: 95–98.
12. Ay F, Bailey TL, Noble WS. Statistical confidence estimation for Hi-C data reveals regulatory chromatin contacts. Genome Res. 2014;24: 999–1011.
13. Di Pierro M, Zhang B, Aiden EL, Wolynes PG, Onuchic JN. Transferable model for chromosome architecture. Proc Natl Acad Sci. 2016;113: 12168–12173.
14. Rycroft CH. VORO++: A three-dimensional Voronoi cell library in C++. Chaos. 2009;19.

Figures.

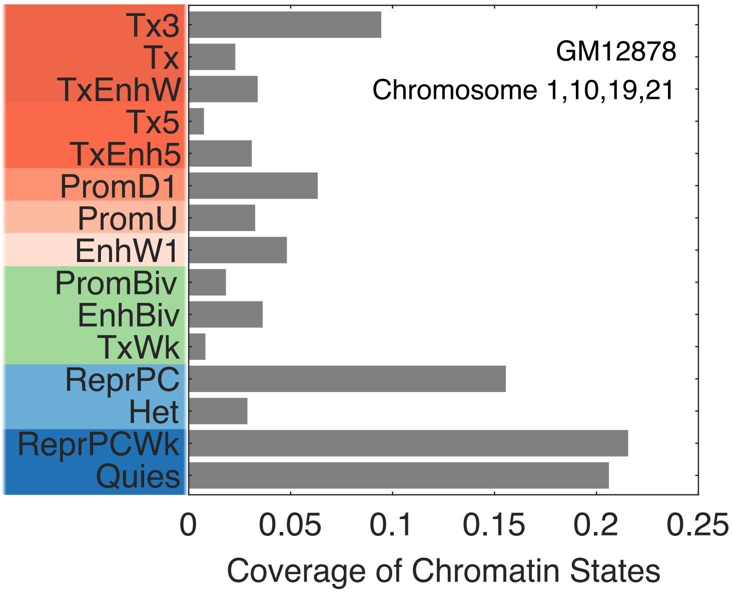

Figure A1: The coverage of various chromatin states in the selected segments of chromosomes 1, 10, 19, and 21 from GM12878 cells.

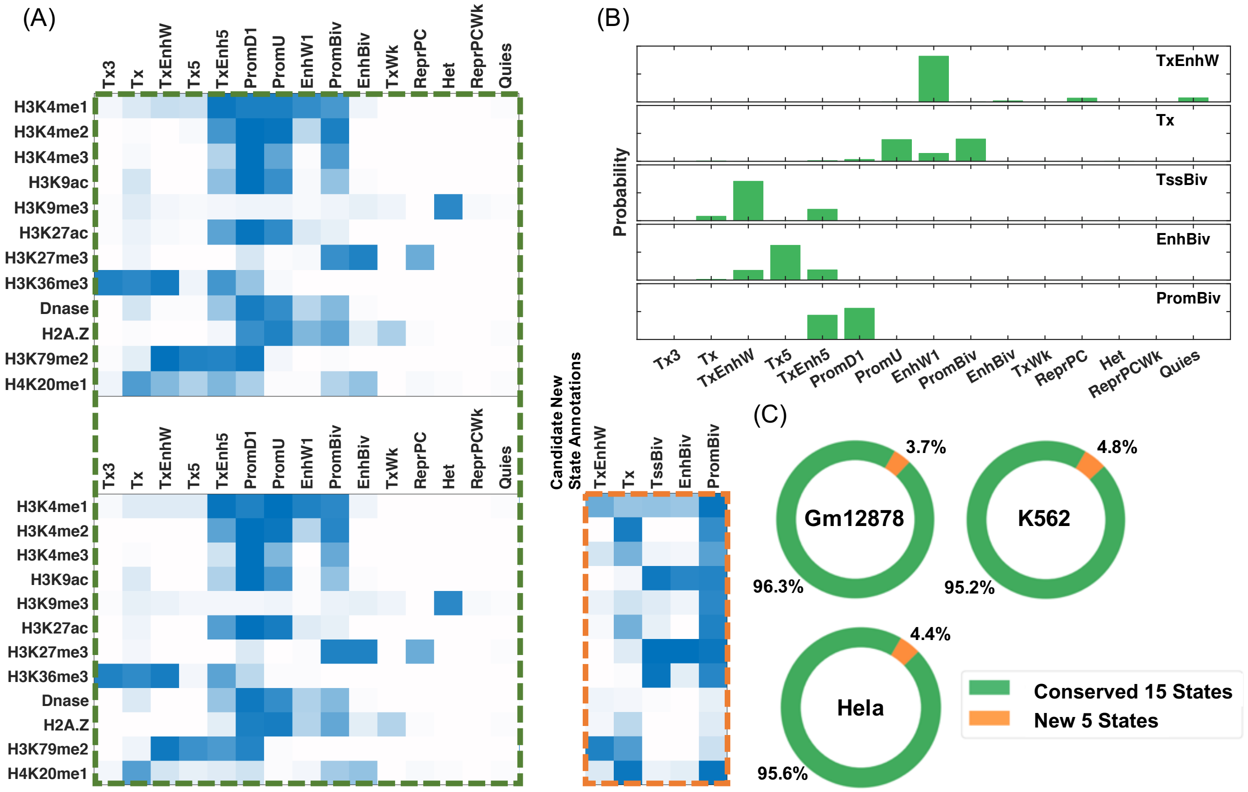

Figure A2: **Comparison between chromatin states used in our model with the ones obtained from a 20-state model.** (A, green) Histone mark patterns for the 15 chromatin states used in the current model (Top) and the 15 states obtained from a new calculation using ChromHMM in which the total number of states is set as 20. It's evident that the two set of states are in good agreement with each other. Histone mark profiles for the additional 5 states are shown in the bottom right. (B) Correspondence between the 5 extra states and the 15 states used in current model. The y-axis indicates the probability that the five new states are assigned to the different chromatin states used in our model. Together with panel A, these data suggest that the five states do not lead to the discovery of novel epigenetic classes, but only provide a fine division of existing enhancer, promoter and transcription states. (C) The additional 5 states only cover 3.7%, 4.8%, 4.4% of the whole genome for Gm12878, K562 and Hela cells, respectively.

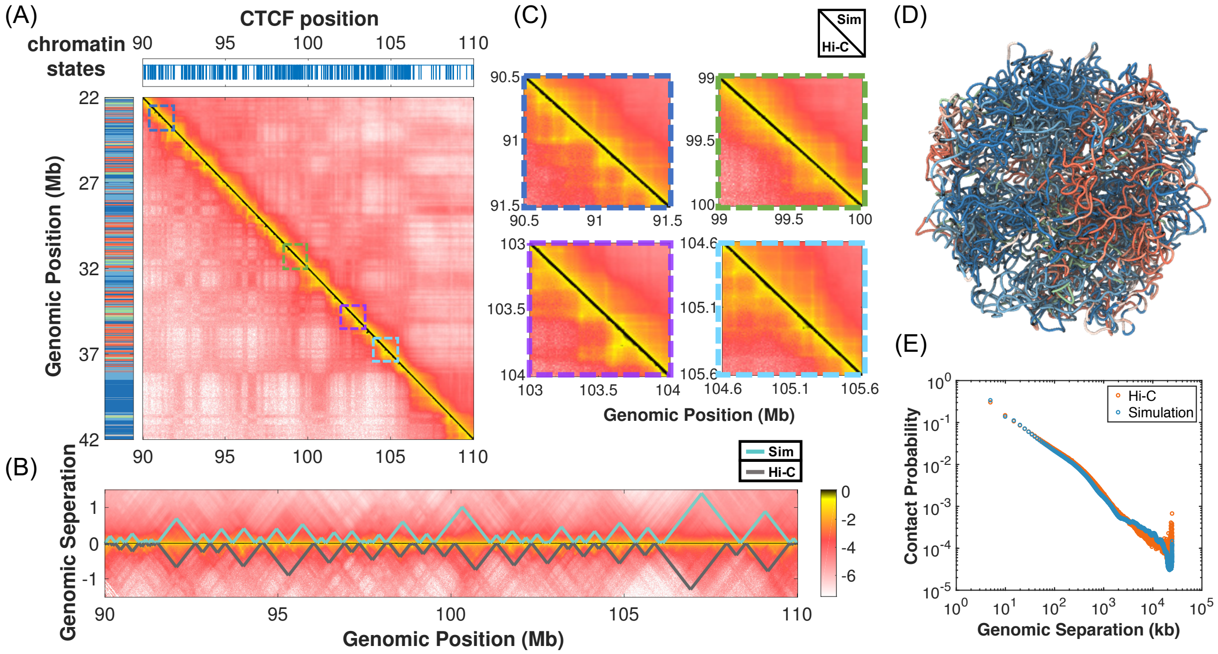

Figure B1: **Comparison between simulated and experimental contact probability maps for a 20 Mb segment of chromosome 10 from GM12878 cells.** (A) Simulated and experimental results are shown in the upper and lower triangle respectively on a log scale. Also shown on the left and top panels are the sequence of chromatin states and the genomic positions of CTCF binding sites. (B) A zoomed-in view of the contact maps along the diagonal region to highlight the formation of TADs. TAD boundaries detected using the software TADbit are plotted on the top of the contact map, with the simulation shown in cyan and experiment in grey. (C) Zoomed-in view of several representative regions along the diagonal to highlight the formation of chromatin loops. (D) A representative chromatin structure predicted by the computational model is drawn in a tube representation and colored by chromatin states. (E) The average contact probability as a function of the genomic separation is shown below on a log-log scale for the simulated (blue) and experimental (red) contact maps respectively.

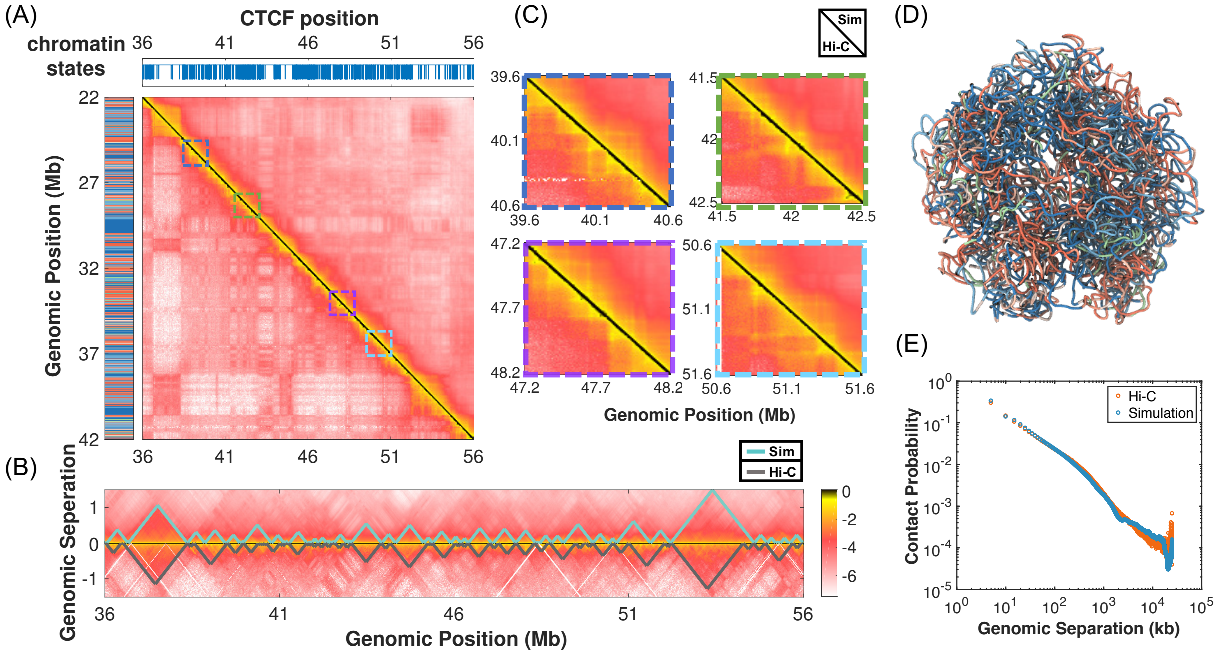

Figure B2: **Comparison between simulated and experimental contact probability maps for a 20 Mb segment of chromosome 19 from GM12878 cells.** (A) Simulated and experimental results are shown in the upper and lower triangle respectively on a log scale. Also shown on the left and top panels are the sequence of chromatin states and the genomic positions of CTCF binding sites. (B) A zoomed-in view of the contact maps along the diagonal region to highlight the formation of TADs. TAD boundaries detected using the software TADbit are plotted on the top of the contact map, with the simulation shown in cyan and experiment in grey. (C) Zoomed-in view of several representative regions along the diagonal to highlight the formation of chromatin loops. (D) A representative chromatin structure predicted by the computational model is drawn in a tube representation and colored by chromatin states. (E) The average contact probability as a function of the genomic separation is shown below on a log-log scale for the simulated (blue) and experimental (red) contact maps respectively.

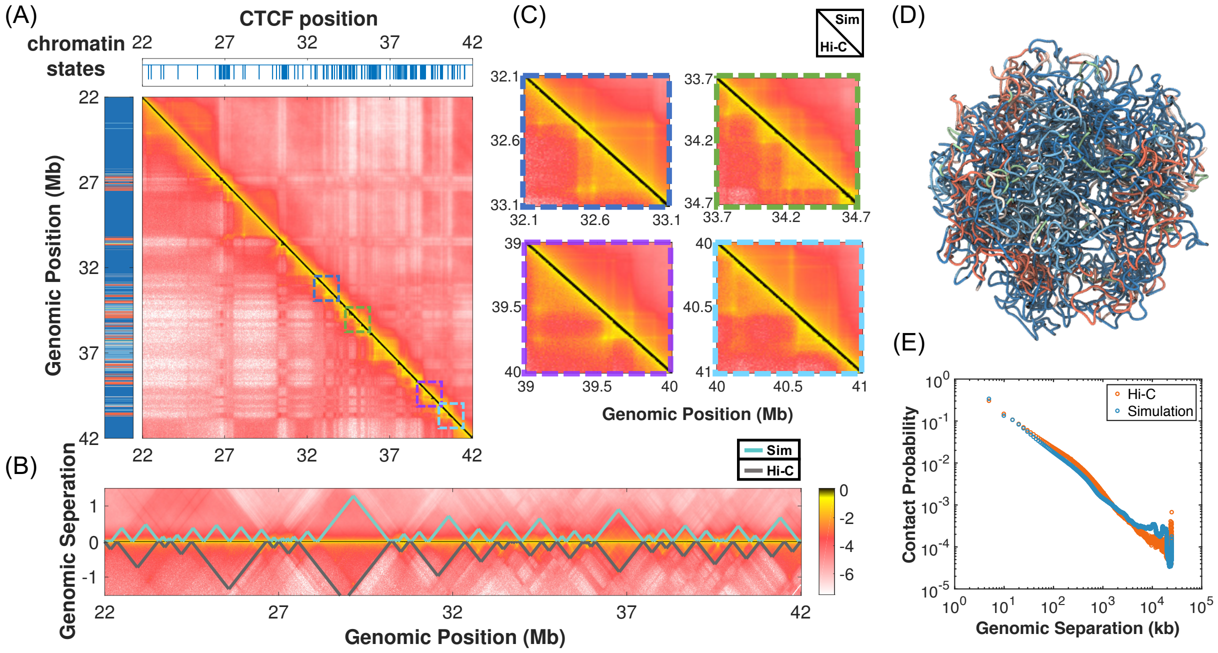

Figure B3: **Comparison between simulated and experimental contact probability maps for a 20 Mb segment of chromosome 21 from GM12878 cells.** (A) Simulated and experimental results are shown in the upper and lower triangle respectively on a log scale. Also shown on the left and top panels are the sequence of chromatin states and the genomic positions of CTCF binding sites. (B) A zoomed-in view of the contact maps along the diagonal region to highlight the formation of TADs. TAD boundaries detected using the software TADbit are plotted on the top of the contact map, with the simulation shown in cyan and experiment in grey. (C) Zoomed-in view of several representative regions along the diagonal to highlight the formation of chromatin loops. (D) A representative chromatin structure predicted by the computational model is drawn in a tube representation and colored by chromatin states. (E) The average contact probability as a function of the genomic separation is shown below on a log-log scale for the simulated (blue) and experimental (red) contact maps respectively.

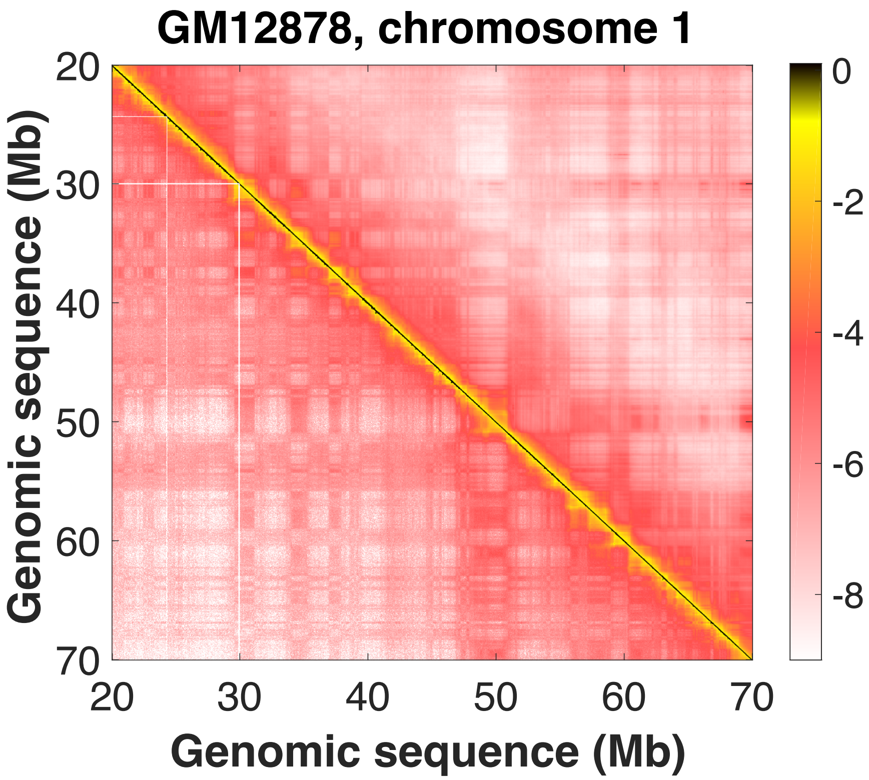

Figure C: Comparison between simulated (top right) and experimental (bottom left) contact probability maps for a 50 Mb segment of chromosome 1 from GM12878 cells. The good agreement between the two suggests that our model can be applied to simulate whole chromosomes.

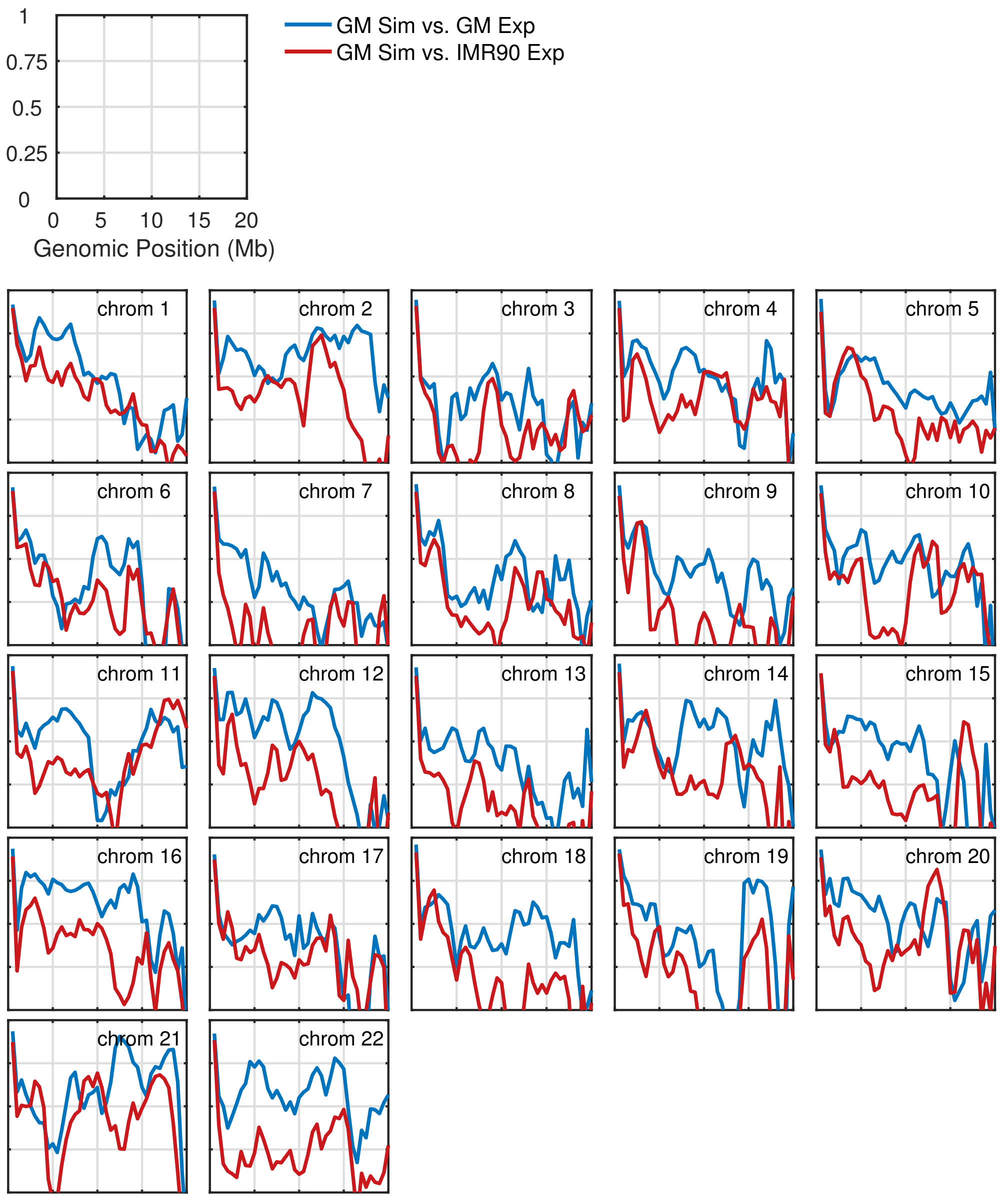

Figure D: Pearson correlation coefficients between simulated and experimental GM12878 Hi-C data (blue) and between simulated GM12878 and experimental IMR90 Hi-C data (red) as a function of genomic separation for different chromosomes. The contact maps constructed at the 50kb resolution were used for these calculations, and the correlation coefficients were calculated with a 500kb increment.

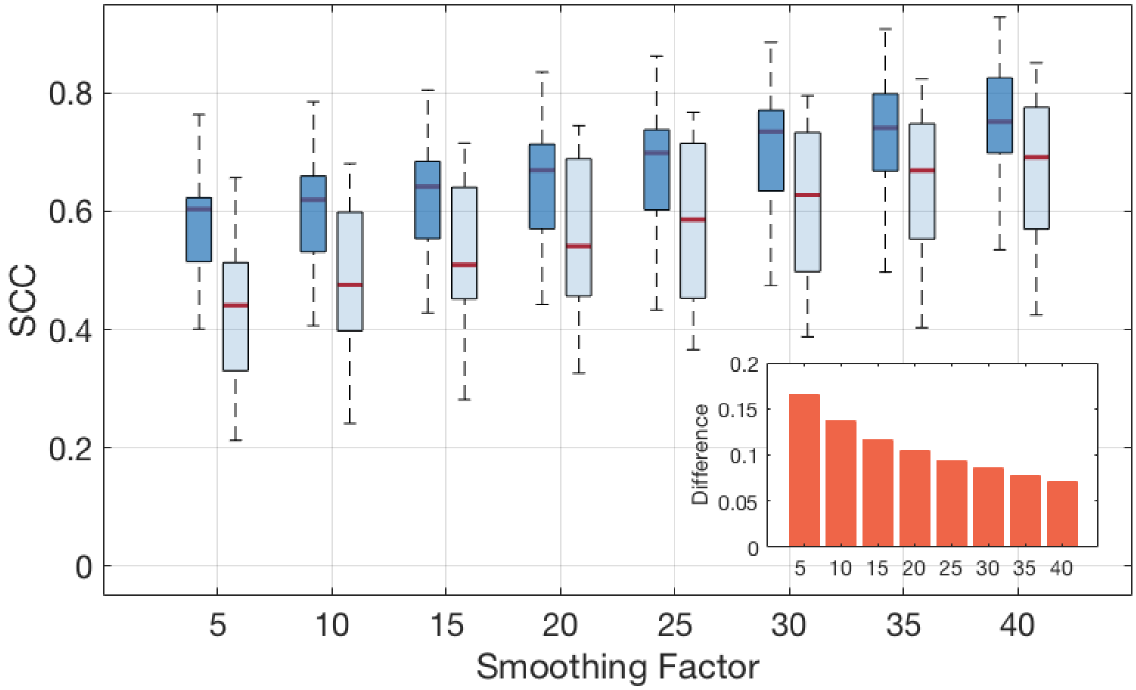

Figure E: **Sensitivity of SCC values with respect to the smoothing parameter used in their calculations.** SCC between simulated and experimental contact maps for GM12878 cells (darker blue), and between simulated GM12878 and experimental IMR90 contact maps (lighter blue) for all 22 chromosome are calculated with different smoothing factors indicated in x-axis. Inset shows the difference between the average value of the two distributions under each smoothing factor. We emphasize that, for all the smoothing parameters studied here, the SCC values between GM-Sim and GM-Exp are always higher than that between GM-Sim and IMR90-Exp.

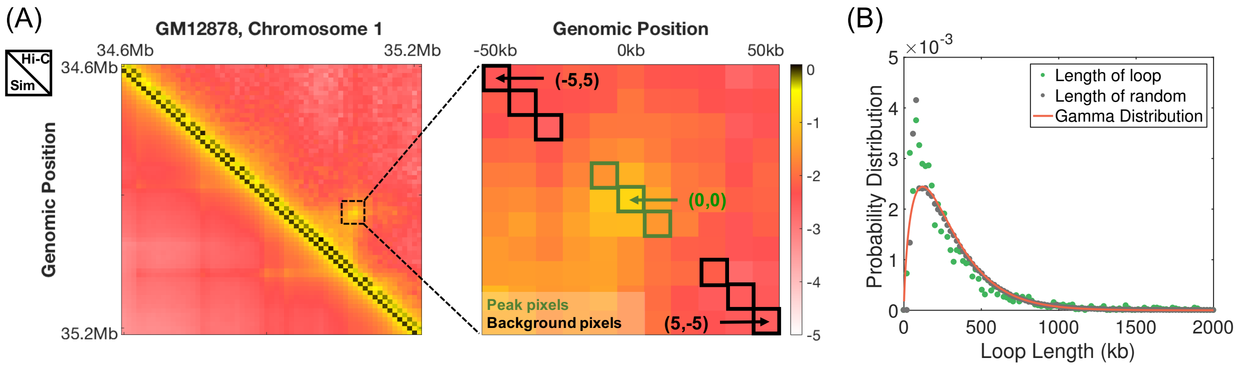

Figure F1: **Quantitative evaluation of the computational model's ability in predicting the formation of chromatin loops.** (A) Illustration for the contact enhancement metric introduced to evaluate the quality of chromatin loop prediction. An example experimental contact probability map (upper triangle) that highlights the formation of a chromatin loop between two genomic loci is shown on the left. The corresponding simulated map is shown in the bottom. A zoomed in view of the map is shown on the right to illustrate the definition of peak and background regions used to calculate the contact enhancement metric. See *SI Section: Contact enhancement metric for chromatin loops* for the definition of contact enhancement. (B) Probability distributions of the genomic separation for chromatin loops identified from Hi-C contact maps (green) and for randomly selected pairs (grey). The orange line is a numerical fit to the chromatin loop length distribution with a Gamma function.

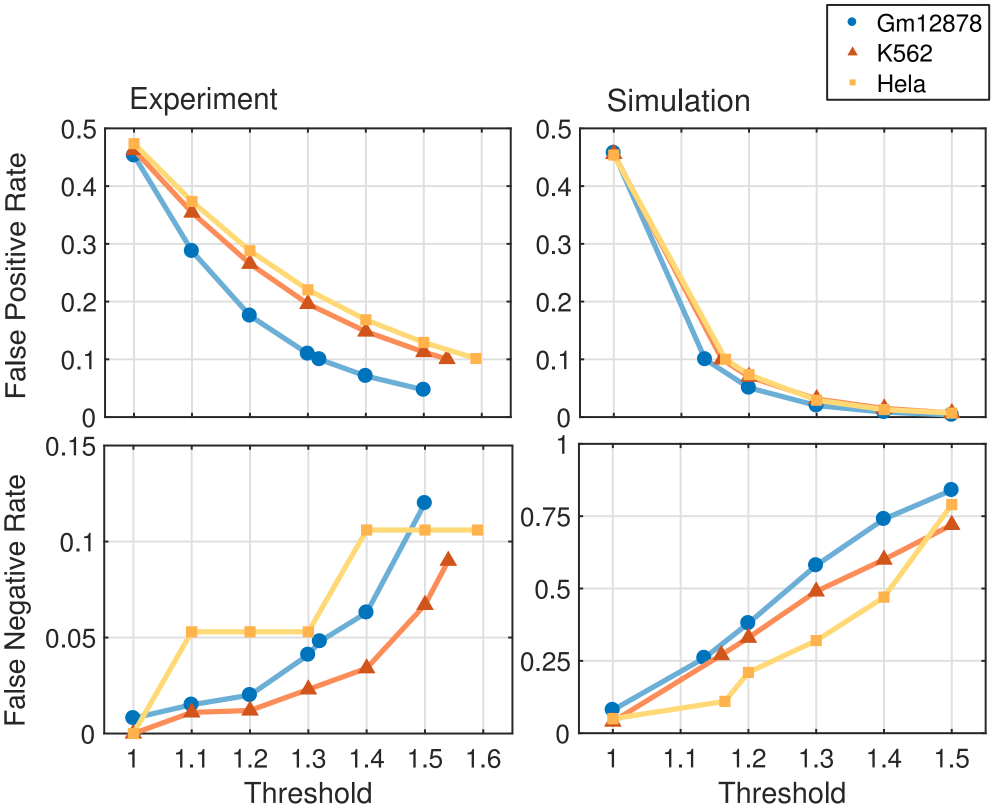

Figure F2: **False positive and negative values in predicting chromatin loops with convergent CTCF pairs using the contact enhancement metric determined with experimental (left) and simulated (right) data.** False positive values are determined as the fraction of random loops with contact enhancement larger than a preset cutoff (x-axis). These random loops, by definition, are not identified as chromatin loops in Hi-C data. On the other hand, false negative values are determined as the fraction of chromatin loops with contact enhancement less than a preset cutoff.

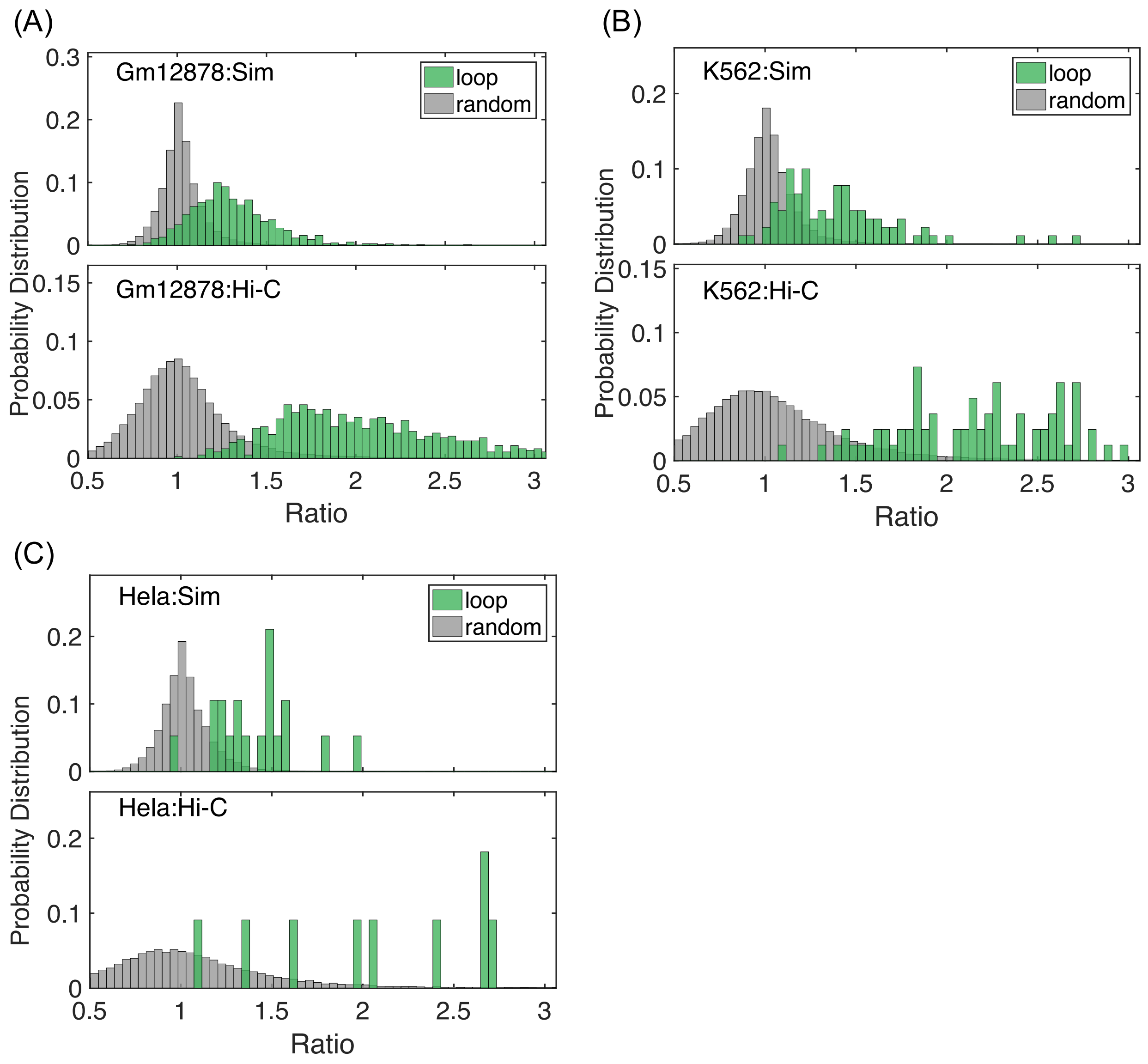

Figure F3: Probability distributions of the contact enhancement for chromatin loops (green) and random pairs (grey) determined from the simulated (top) and the Hi-C contact maps (bottom) for GM12878 (A), K562 (B) and Hela cells (C). Chromatin loops with CTCF molecules in a convergent orientation at the loop anchors and contact maps from chromosomes 1 to 22 were used to calculate these distributions.

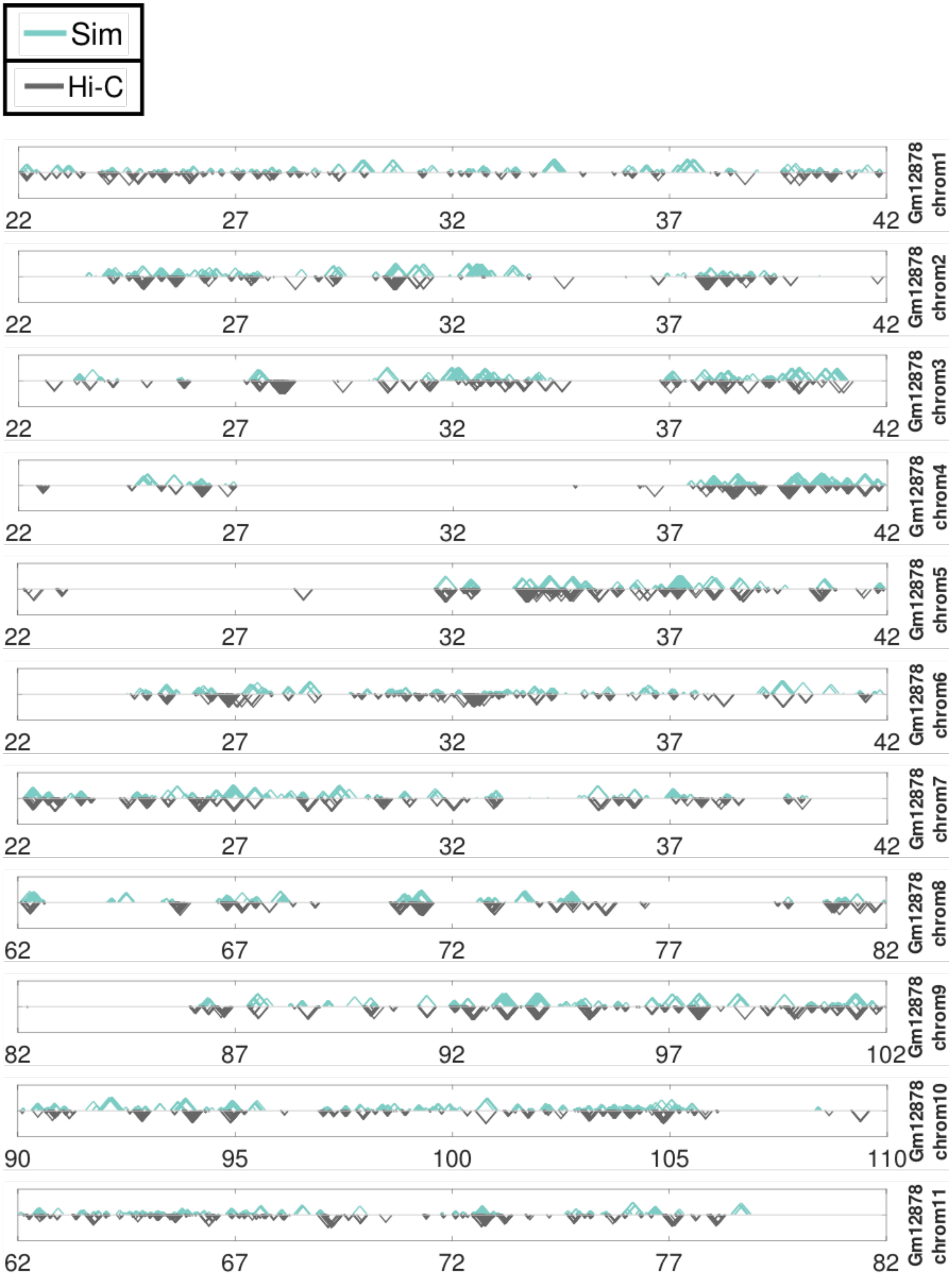

Figure G1: **Comparison between significant contact pairs determined from simulated and experimental contact probability maps using the software FitHiC.** See text *SI Section: De novo detection of loops and TADs* for detailed explanations.

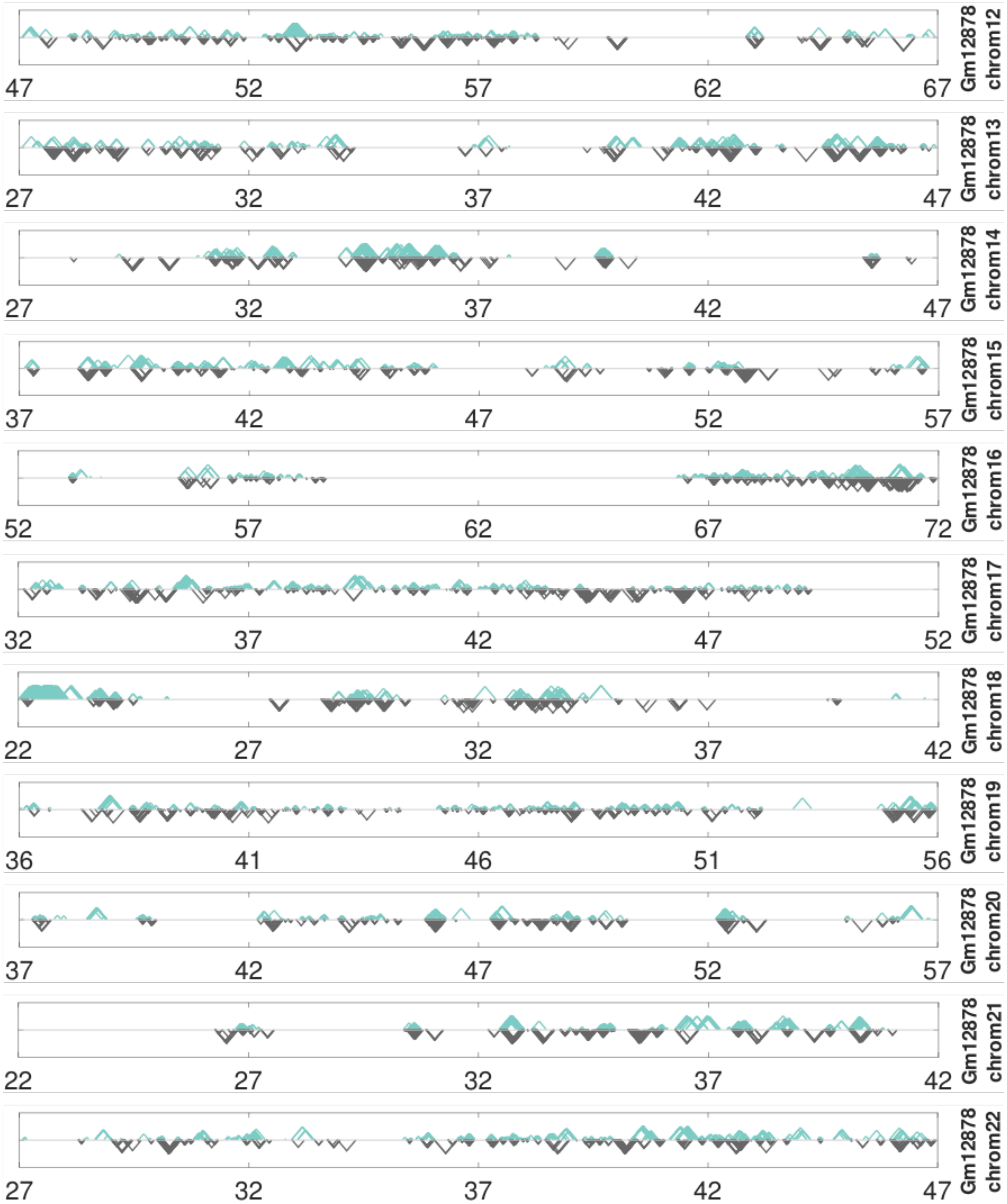

Figure G2: **Continuation of Figure S6-1 for the rest of chromosomes.**

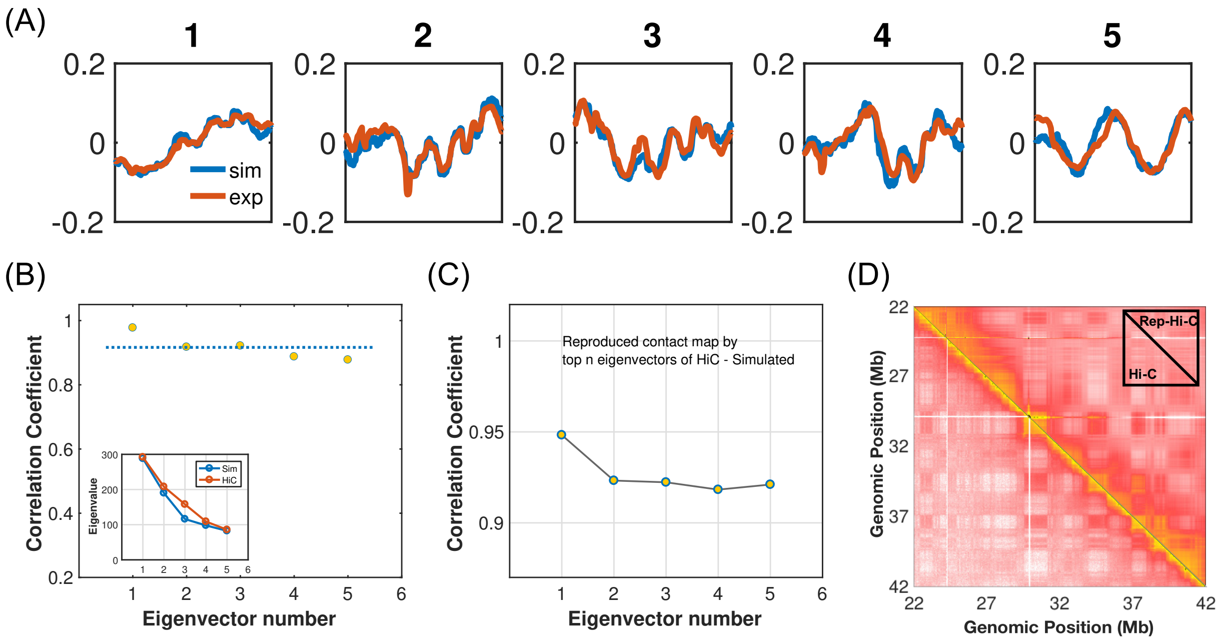

Figure H1: **Eigenvector analysis of the contact probability maps for chromosome 1 from GM12878 cells.** (A) Top five eigenvectors for simulated (blue) and experimental (orange) log contact matrices. (B) Correlation coefficients for the eigenvectors shown in (A). The average correlation coefficient (dotted line) is 0.917. The inset shows the comparison of the top five eigenvalues. (C) Correlation coefficients for the reproduced contact maps by the top n eigenvectors of the experimental and simulated contact map as a function of n. (D) Contact map reconstructed using top five eigenvectors (top right) recapitulates the formation of TADs and compartments observed in the original map (bottom left).

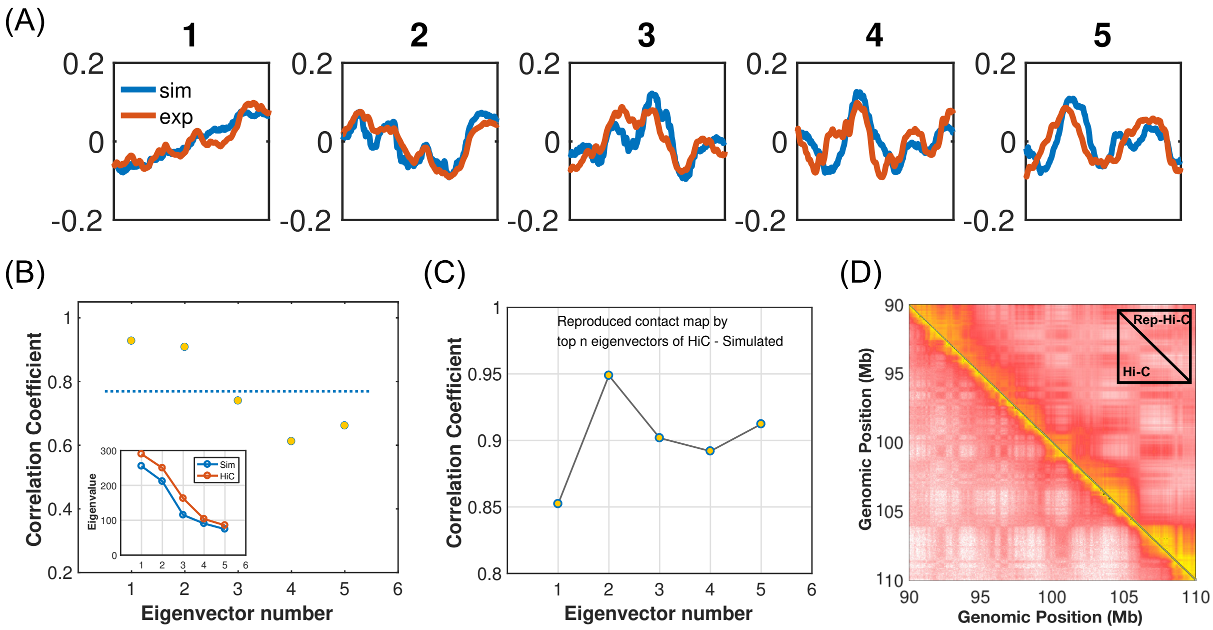

Figure H2: **Eigenvector analysis of the contact probability maps for chromosome 10 from GM12878 cells.** (A) Top five eigenvectors for simulated (blue) and experimental (orange) log contact matrices. (B) Correlation coefficients for the eigenvectors shown in (A). The average correlation coefficient (dotted line) is 0.770. The inset shows the comparison of the top five eigenvalues. (C) Correlation coefficients for the reproduced contact maps by the top n eigenvectors of the experimental and simulated contact map as a function of n. (D) Contact map reconstructed using top five eigenvectors (top right) recapitulates the formation of TADs and compartments observed in the original map (bottom left).

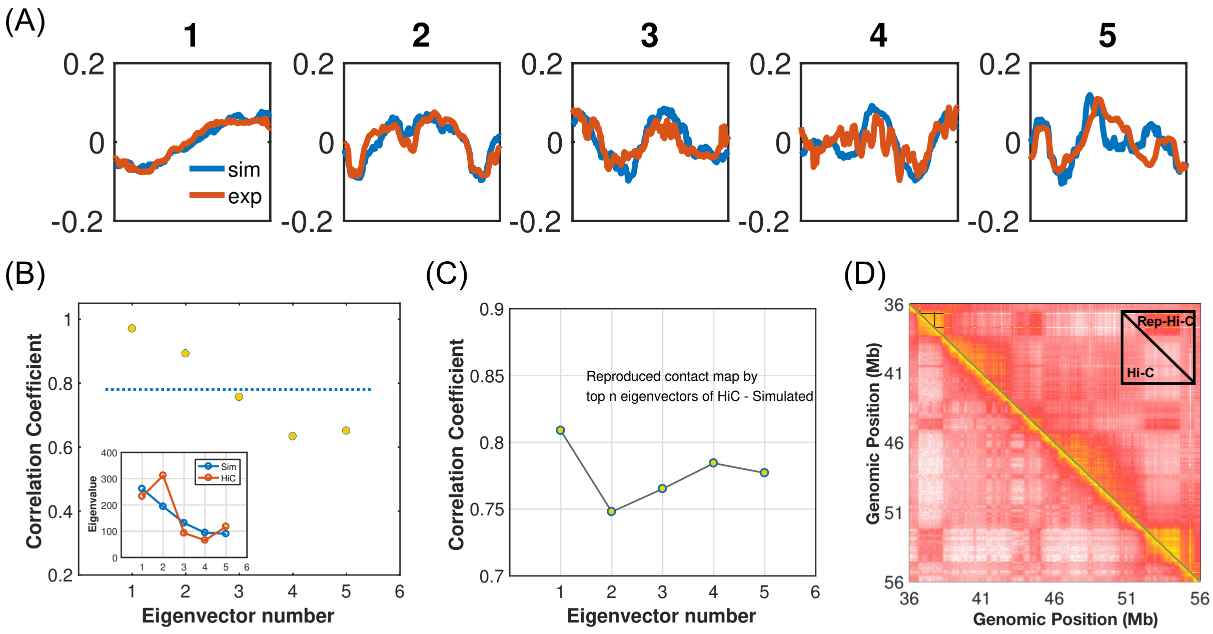

Figure H3: **Eigenvector analysis of the contact probability maps for chromosome 19 from GM12878 cells.** (A) Top five eigenvectors for simulated (blue) and experimental (orange) log contact matrices. (B) Correlation coefficients for the eigenvectors shown in (A). The average correlation coefficient (dotted line) is 0.781. The inset shows the comparison of the top five eigenvalues. (C) Correlation coefficients for the reproduced contact maps by the top n eigenvectors of the experimental and simulated contact map as a function of n. (D) Contact map reconstructed using top five eigenvectors (top right) recapitulates the formation of TADs and compartments observed in the original map (bottom left).

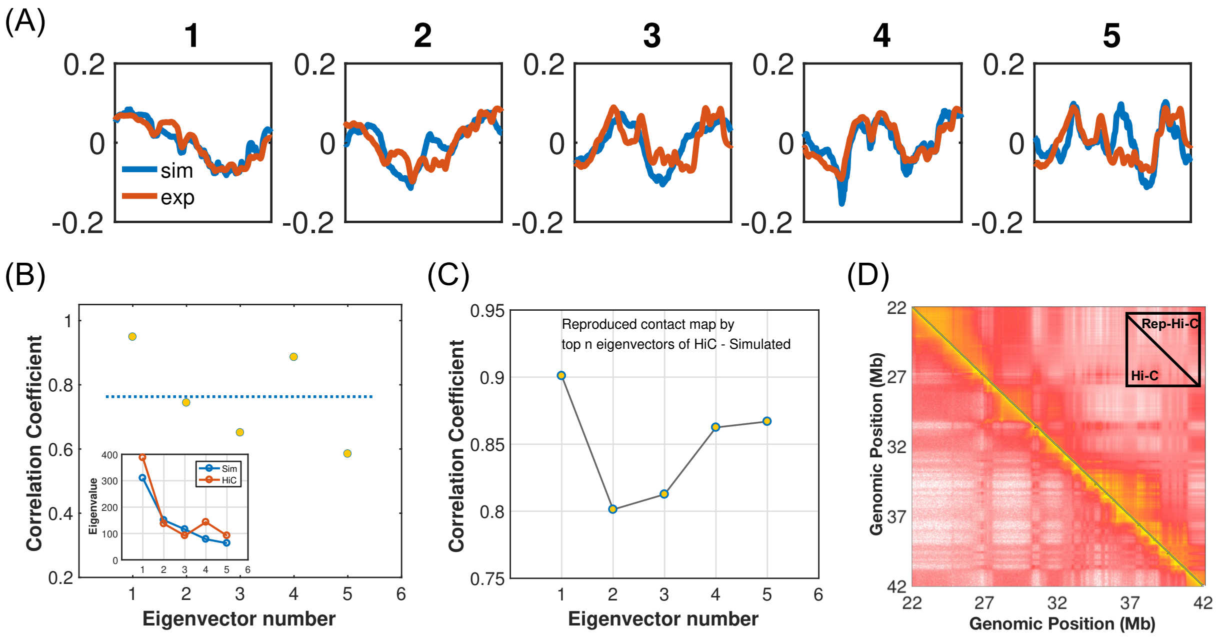

Figure H4: **Eigenvector analysis of the contact probability maps for chromosome 21 from GM12878 cells.** (A) Top five eigenvectors for simulated (blue) and experimental (orange) log contact matrices. (B) Correlation coefficients for the eigenvectors shown in (A). The average correlation coefficient (dotted line) is 0.763. The inset shows the comparison of the top five eigenvalues. (C) Correlation coefficients for the reproduced contact maps by the top n eigenvectors of the experimental and simulated contact map as a function of n. (D) Contact map reconstructed using top five eigenvectors (top right) recapitulates the formation of TADs and compartments observed in the original map (bottom left).

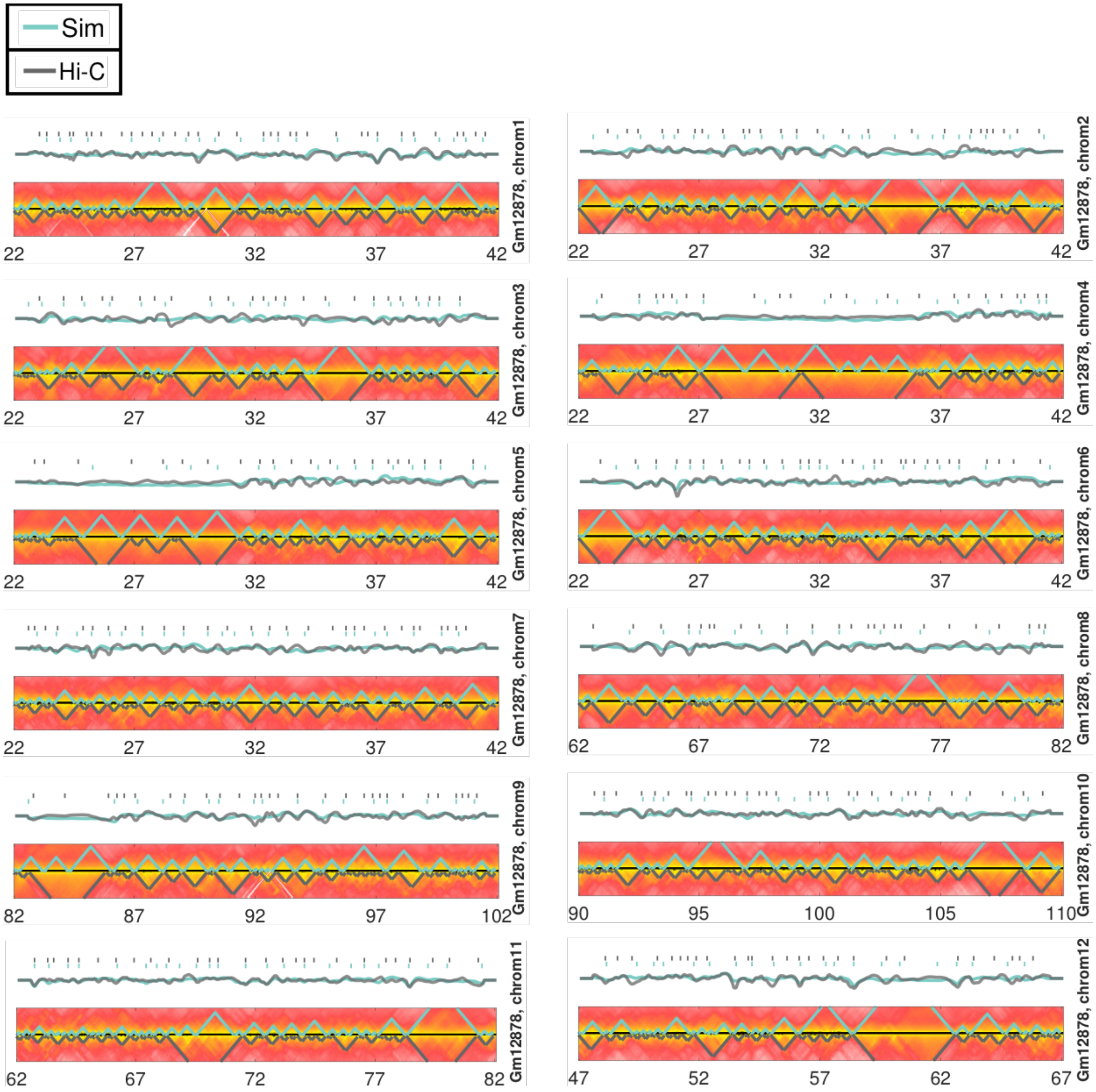

Figure I1: **Comparison between TAD boundaries determined from simulated and experimental contact probability maps for GM12878 cells.** The boundaries detected using the software TADbit are plotted on top of the contact map, with the simulation shown in cyan and experiment in grey. The insulation scores are shown as continuous lines and their corresponding minima are indicated as dots above.

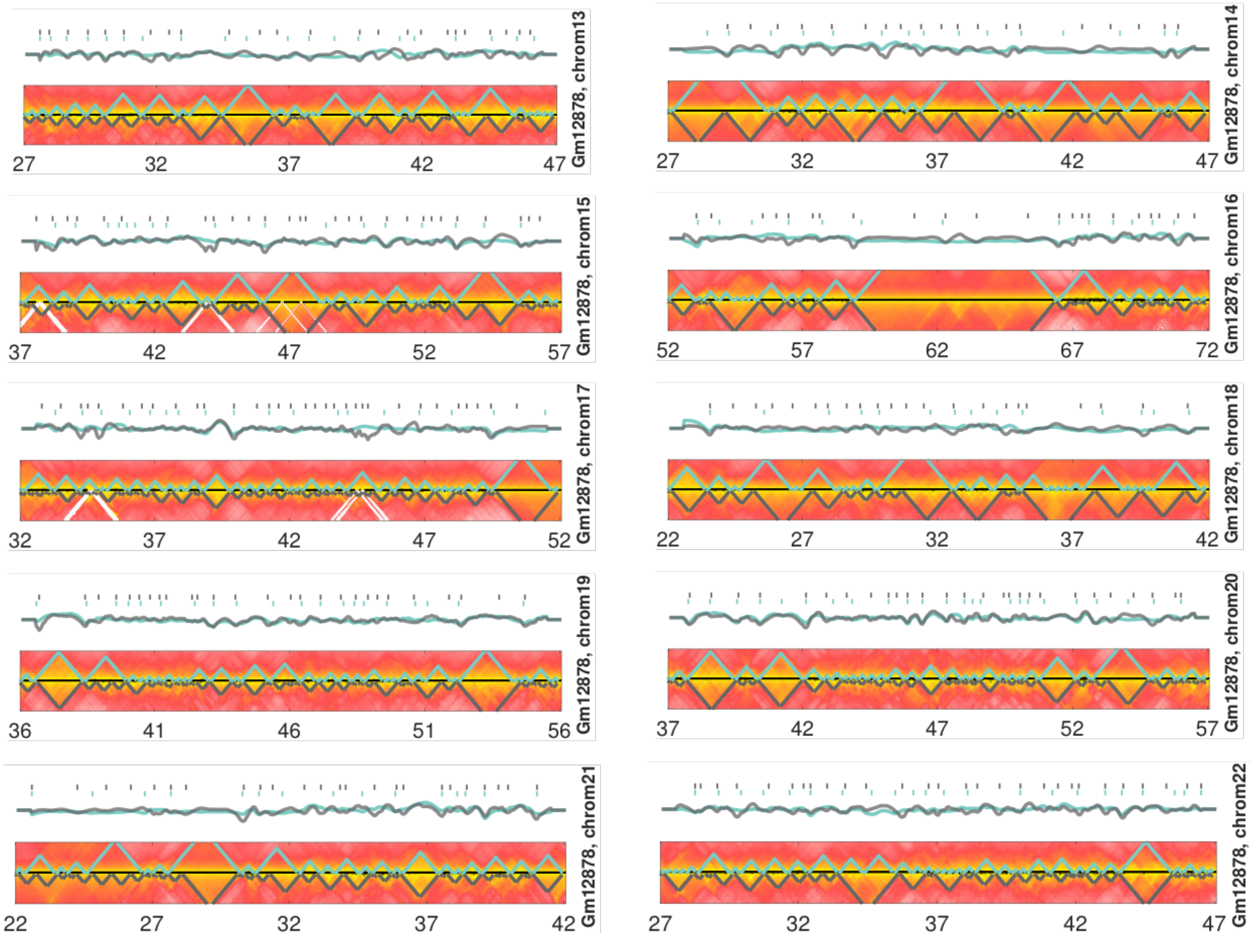

Figure I2: Continuation of Figure S8-1 for the rest of chromosomes.

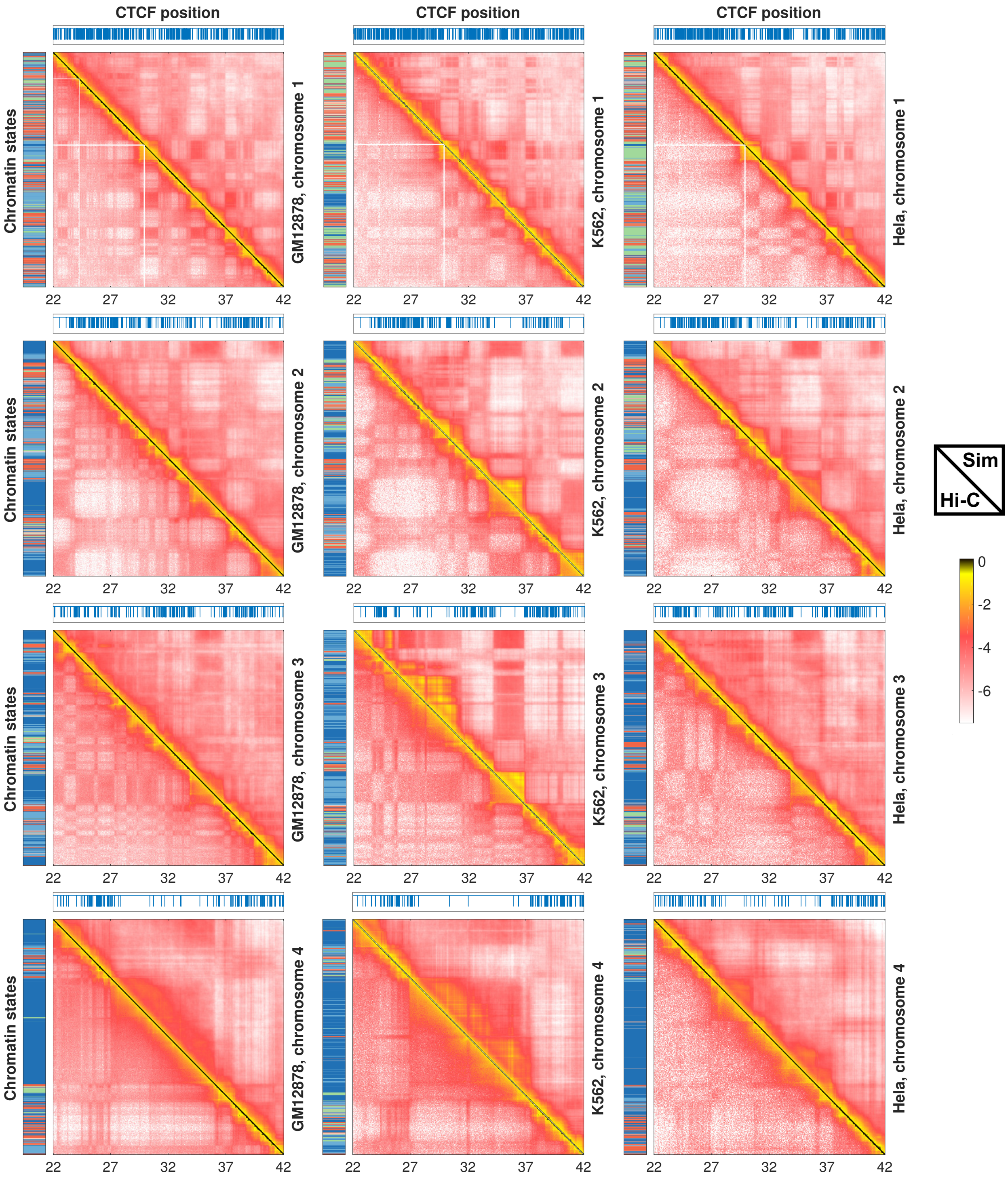

Figure J1: **Comparison between experimental (bottom left) and simulated (top right) contact maps for chromosomes 1-4 from GM12878 (left), K562 (middle) and Hela (right) cells.** Also shown on the left and top panels are the sequence of chromatin states and the genomic positions of CTCF binding sites.

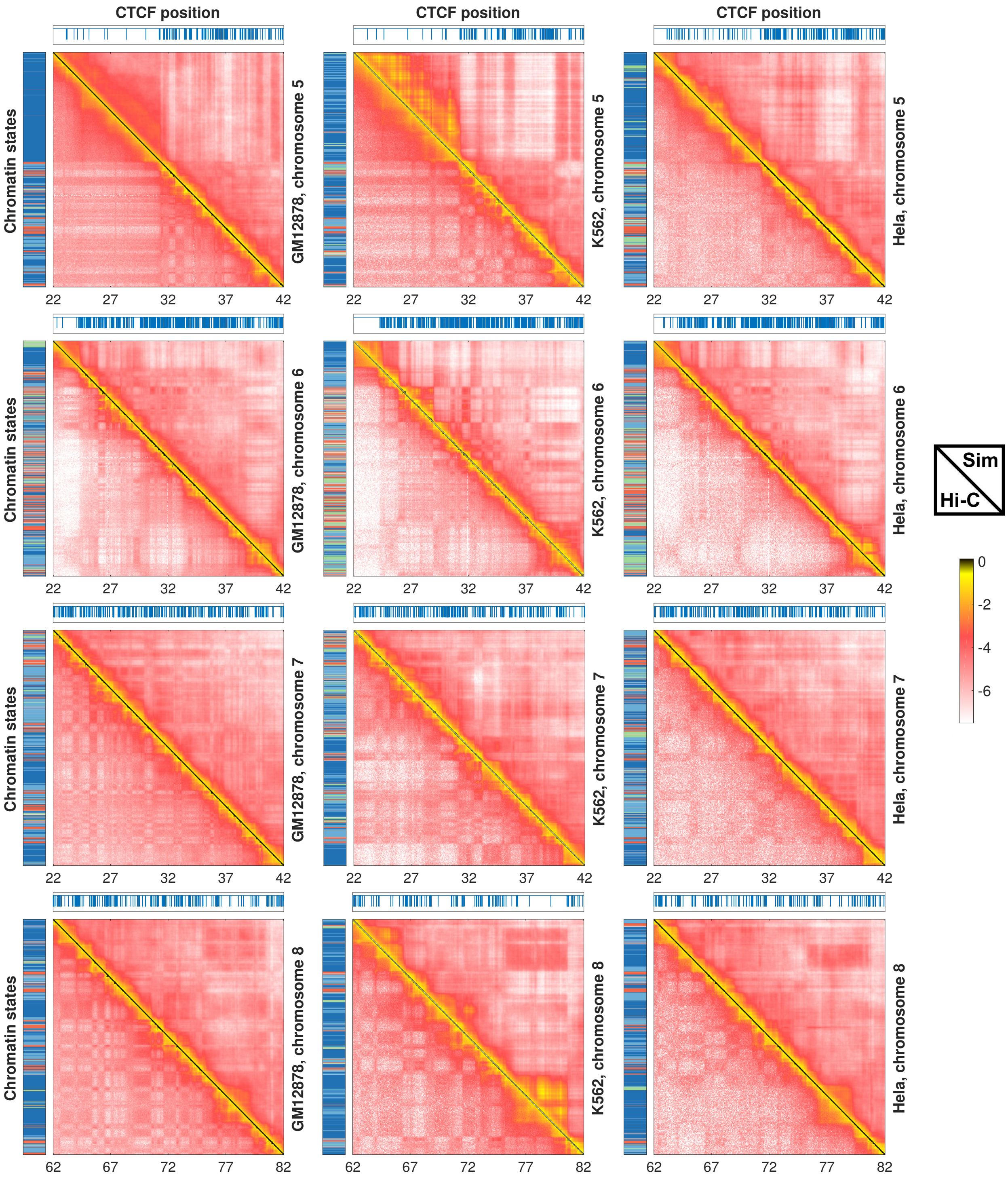

Figure J2: **Comparison between experimental (bottom left) and simulated (top right) contact maps for chromosomes 5-8 from GM12878 (left), K562 (middle) and Hela (right) cells.** Also shown on the left and top panels are the sequence of chromatin states and the genomic positions of CTCF binding sites.

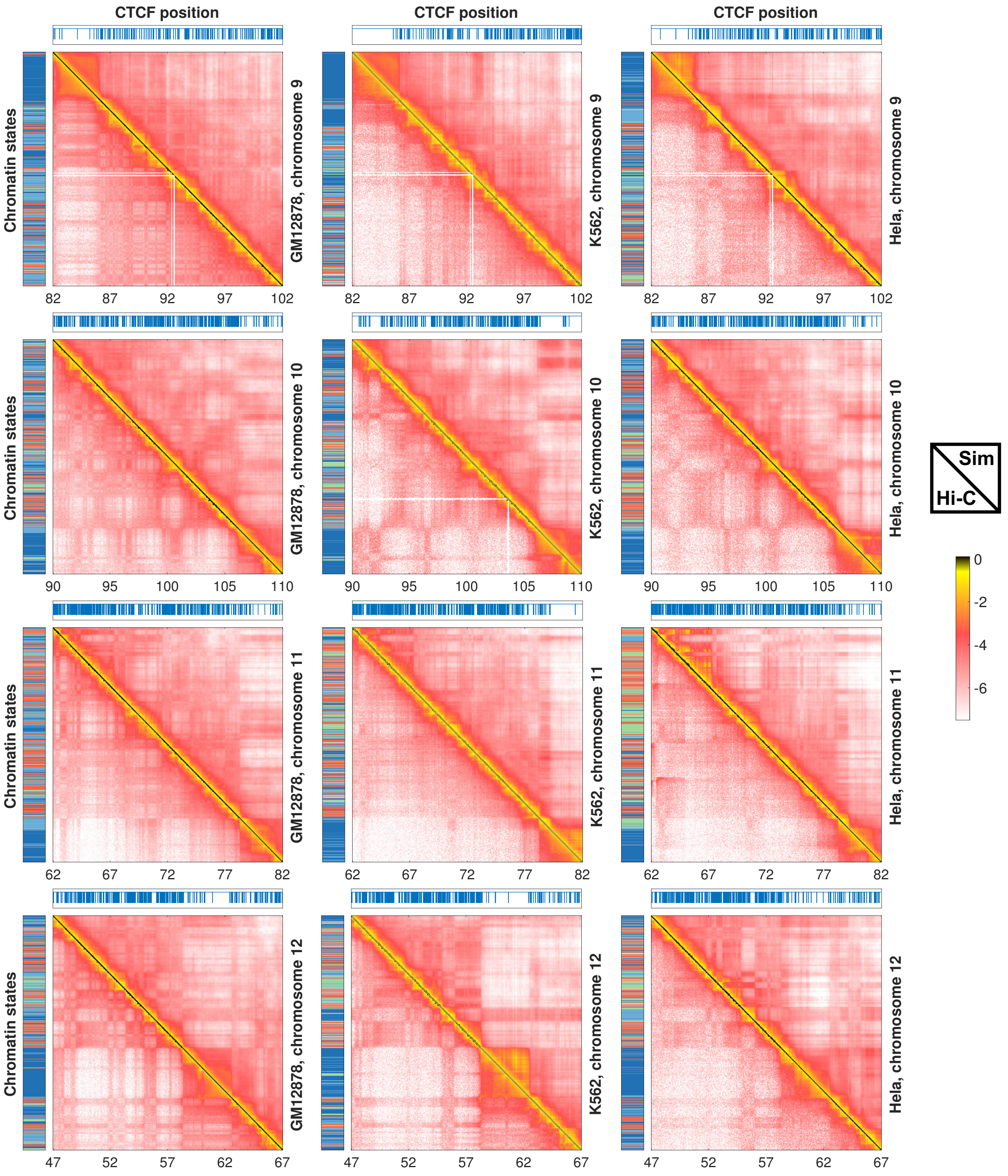

Figure J3: Comparison between experimental (bottom left) and simulated (top right) contact maps for chromosomes 9-12 from GM12878 (left), K562 (middle) and Hela (right) cells. Also shown on the left and top panels are the sequence of chromatin states and the genomic positions of CTCF binding sites.

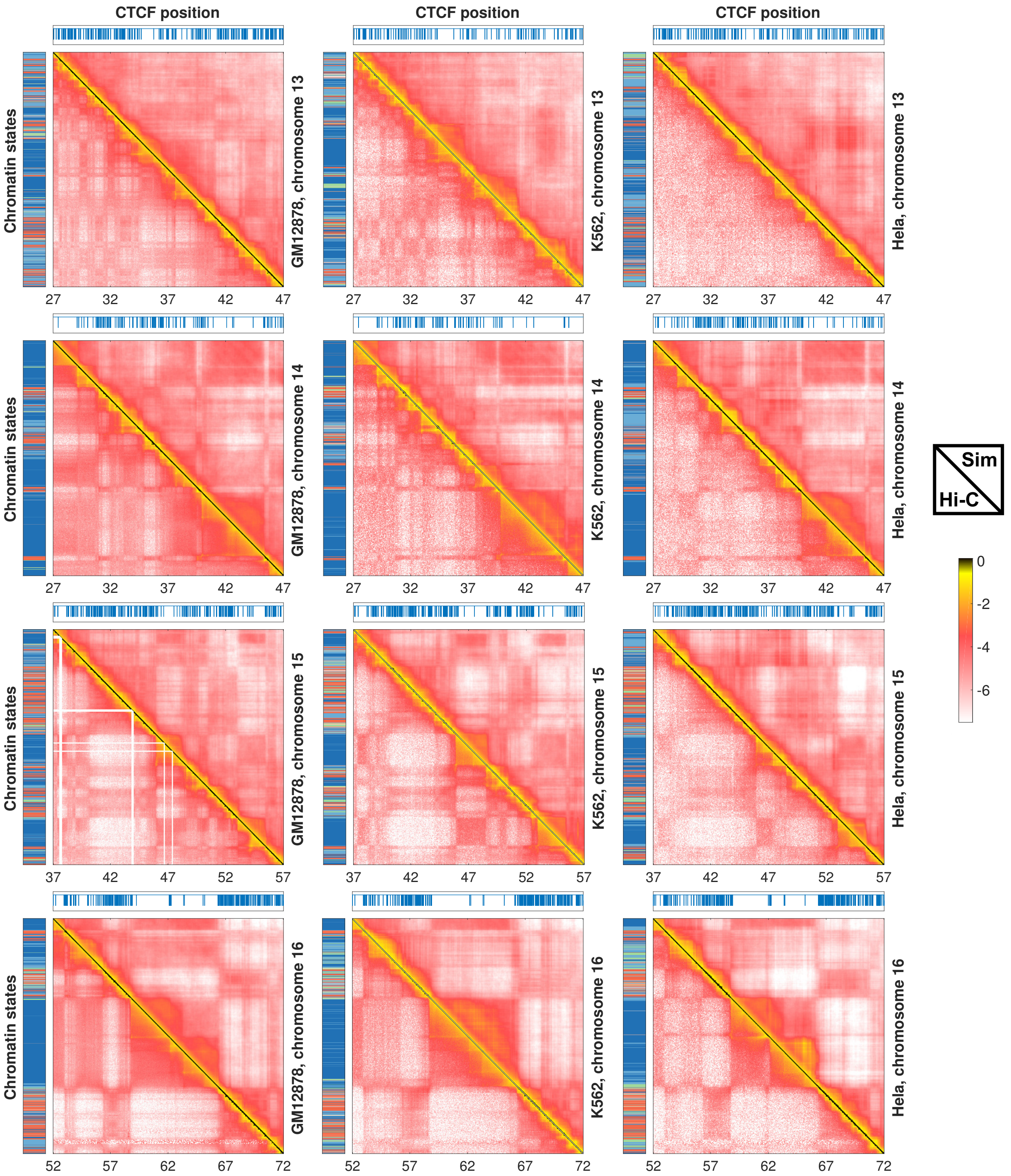

Figure J4: **Comparison between experimental (bottom left) and simulated (top right) contact maps for chromosomes 13-16 from GM12878 (left), K562 (middle) and Hela (right) cells.** Also shown on the left and top panels are the sequence of chromatin states and the genomic positions of CTCF binding sites.

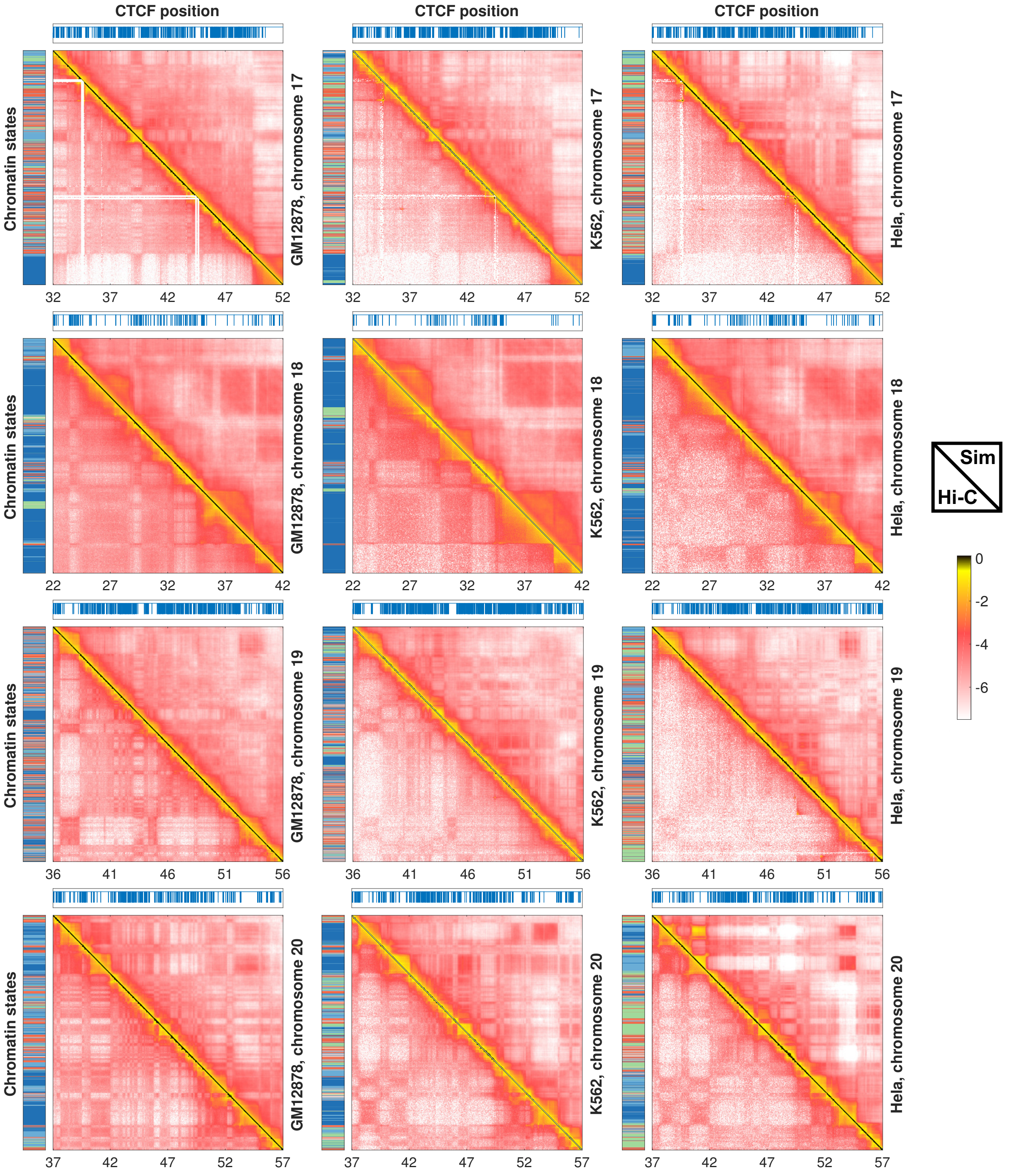

Figure J5: Comparison between experimental (bottom left) and simulated (top right) contact maps for chromosomes 17-20 from GM12878 (left), K562 (middle) and Hela (right) cells. Also shown on the left and top panels are the sequence of chromatin states and the genomic positions of CTCF binding sites.

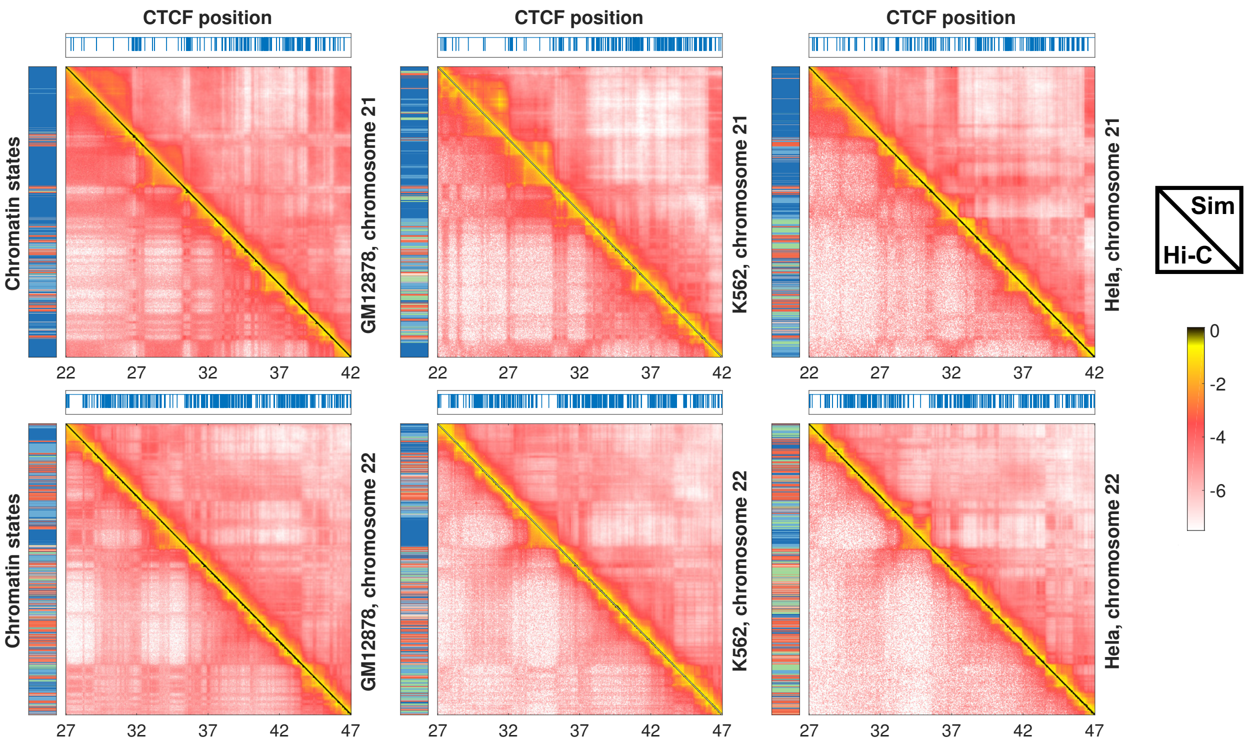

Figure J6: **Comparison between experimental (bottom left) and simulated (top right) contact maps for chromosomes 21-22 from GM12878 (left), K562 (middle) and Hela (right) cells.** Also shown on the left and top panels are the sequence of chromatin states and the genomic positions of CTCF binding sites.

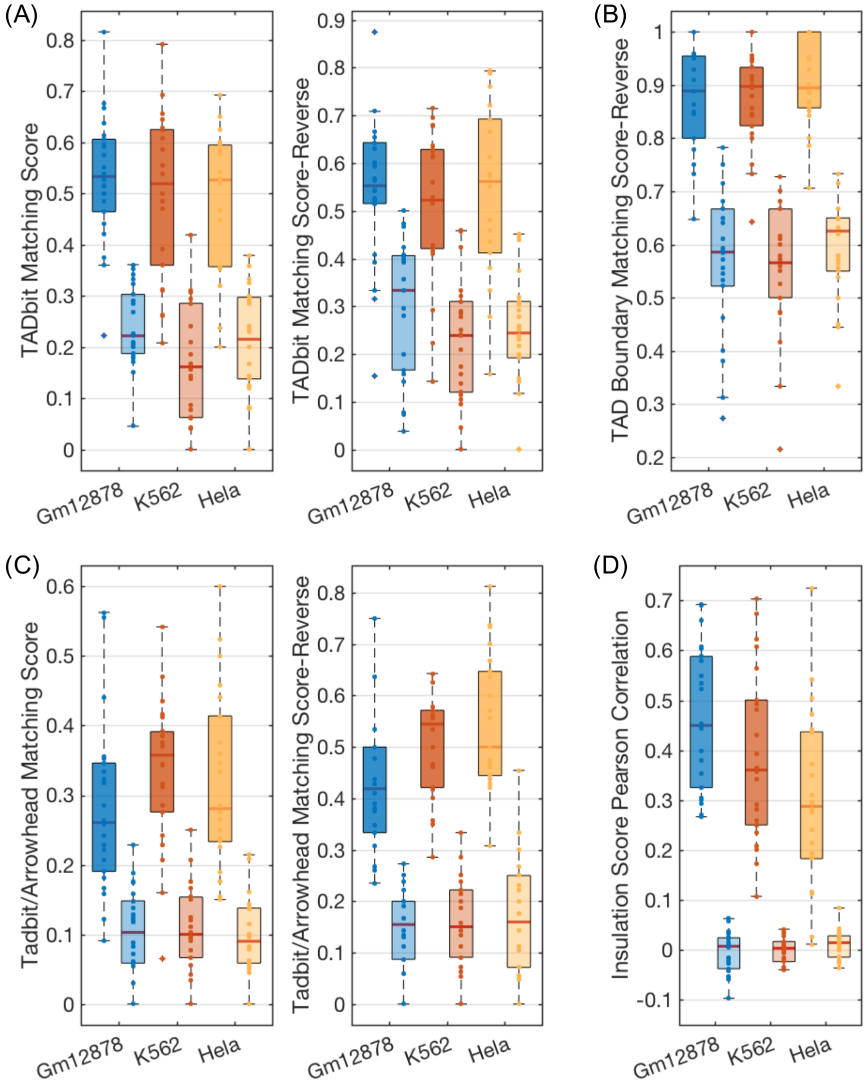

Figure K1: **Quantitative comparison between TADs detected from simulated and experimental contact probability maps.** Matching score for TAD boundaries determined using the software TADbit (A) and from minima of the insulation score profiles (B). The same analysis was applied onto both simulated and experimental data. (C) Comparison between experimental TAD boundaries identified from two software TADbit and Arrowhead. The discrepancy between these results highlight the challenges in robustly inferring TAD boundaries from contact maps. (D) Pearson correlation coefficient of the insulation score profiles derived from the simulated and experimental contact maps. Data shown as light colors in these figures correspond to the analysis between experimental and control data that are obtained by randomly shuffling the size of the TADs along the chromosome while keeping total number unchanged. The boxes represent the 25% and 75% quantities of the matching score distribution, and the thick line inside each box corresponds to the median value. Whiskers indicate the last values that fall within 1.5 times the interquartile range. See text **De novo detection of TADs and significant contacts** for more discussions.

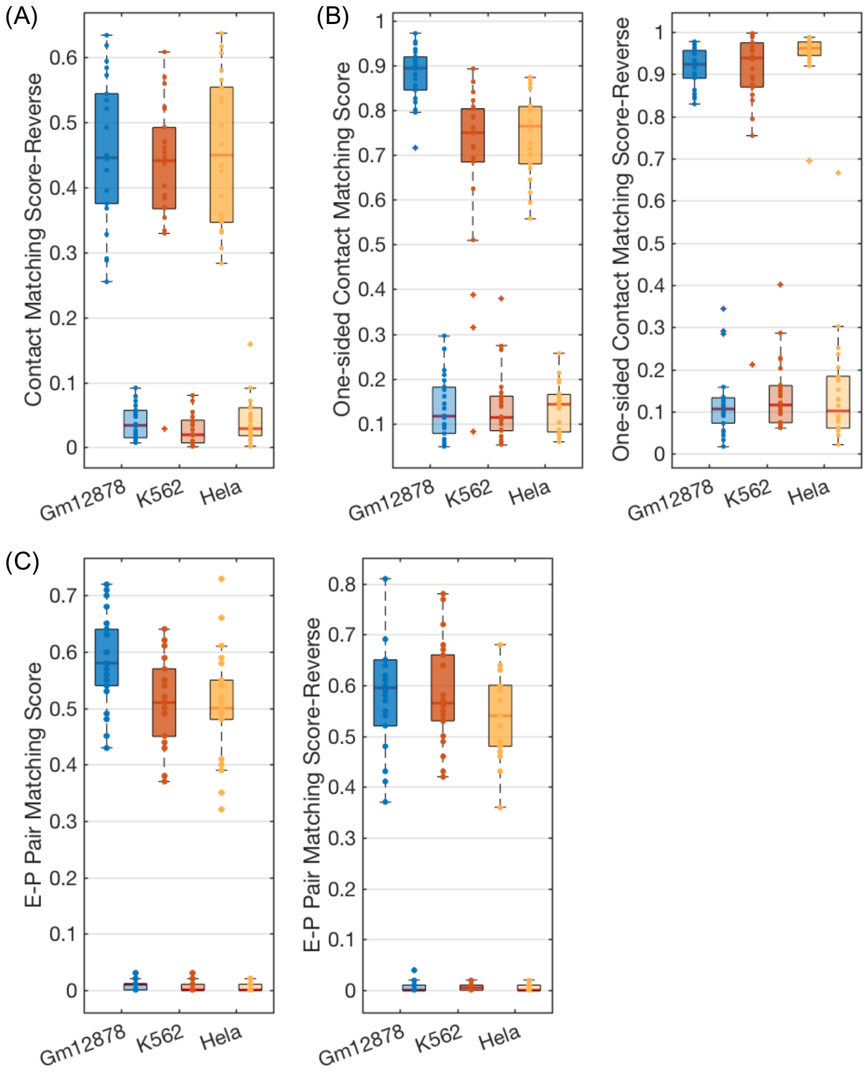

Figure K2: **Quantitative comparison between significant contact pairs and enhancer-promoter pairs detected from simulated and experimental contact probability maps.** Matching score determined using a strict criterion that precisely matches both loop boundaries (A) and using a relaxed criterion that allows a large deviation for one of the loop boundaries (B). (C) Matching score determined using a strict criterion that precisely matches both pair loci for the top 100 enhancer-promoter pairs with the most significant contacts identified from the experimental and simulated contact probability maps respectively. Data shown as light colors in these figures correspond to the analysis between experimental and control data that are obtained by randomly shuffling the size of contact/enhancer-promoter pairs along the chromosome while keeping their total number unchanged. The boxes represent the 25% and 75% quantities of the matching score distribution, and the thick line inside each box corresponds to the median value. Whiskers indicate the last values that fall within 1.5 times the interquartile range. See text **De novo detection of TADs and significant contacts** for more discussions.

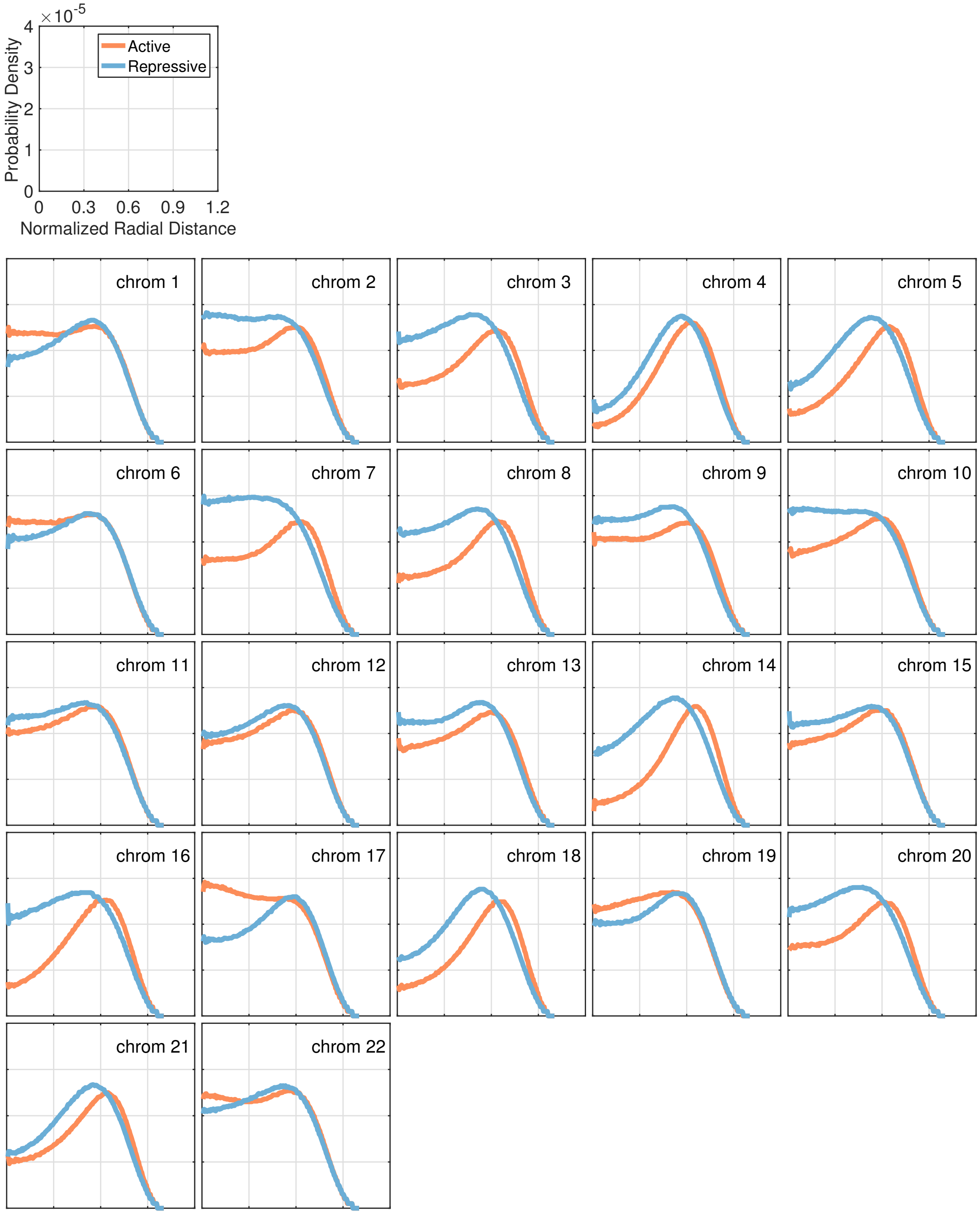

Figure L1: **Radial density profiles of active (states: Tx3, Tx, TxEnhW, Tx5, TxEnh5, PromD1, PromU, EnhW1 in Figure 1) and inactive (states: ReprPC and Het in Figure 1) chromatin states in different chromosomes from GM12878 cells.** The x-axis is normalized by the radii of the confinement that was chosen to ensure a volume fraction 0.1 for chromosomes. The y-axis is further normalized by the number of beads for each chromatin type such that the profiles will integrate to 1.

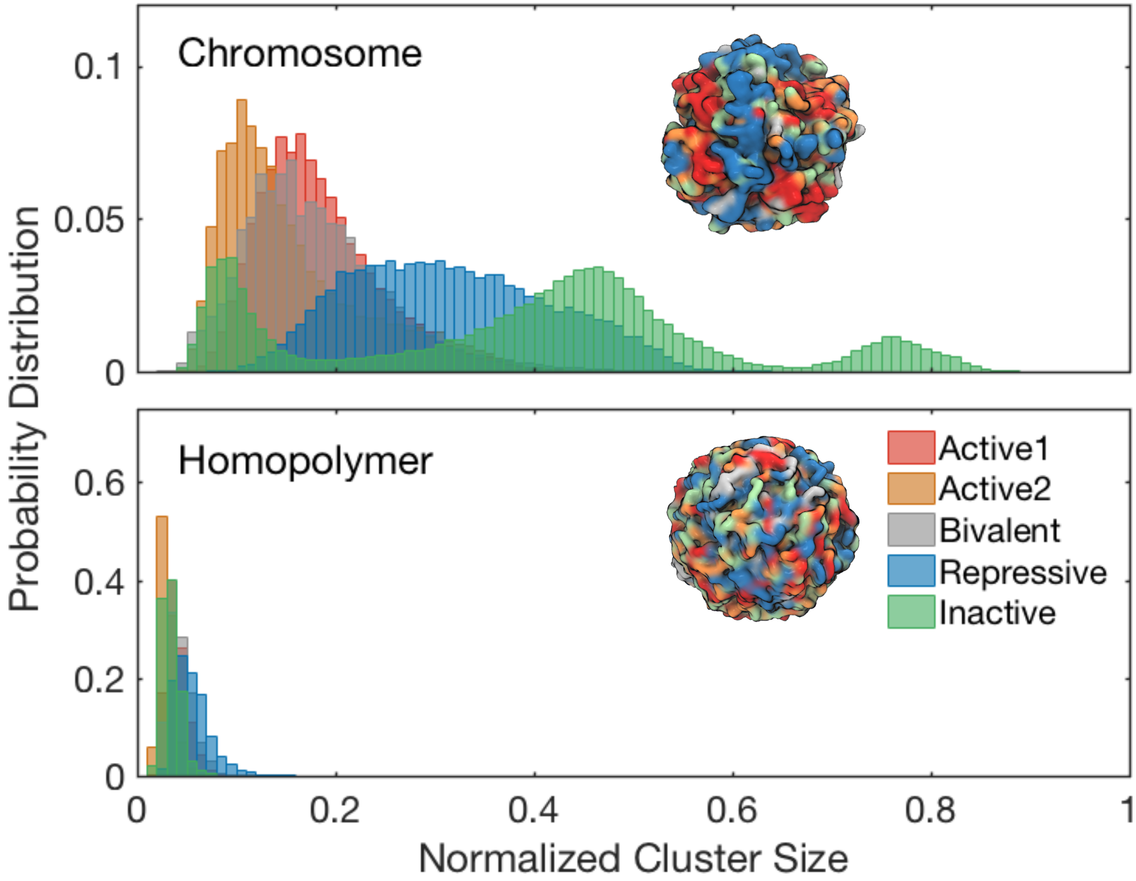

Figure L2: **Clustering analysis of simulated chromosome structures.** The probability distribution of the cluster size for different chromatin types calculated using the structural ensemble for chromosomes 1, 10, 19, 21 from GM12878 cells and for a homopolymer of the same length (Bottom). The largest two connected networks were used to determine the cluster sizes. See *SI Section: Clustering analysis of simulated chromosome structures* for details of the clustering algorithm. Representative chromosome and homopolymer structures colored by chromatin types are shown in the insets with a surface representation. Comparing the results shown in the top and bottom panel, it is clear that there is a tendency for genomic loci of the same chromatin type to co-localize spatially.

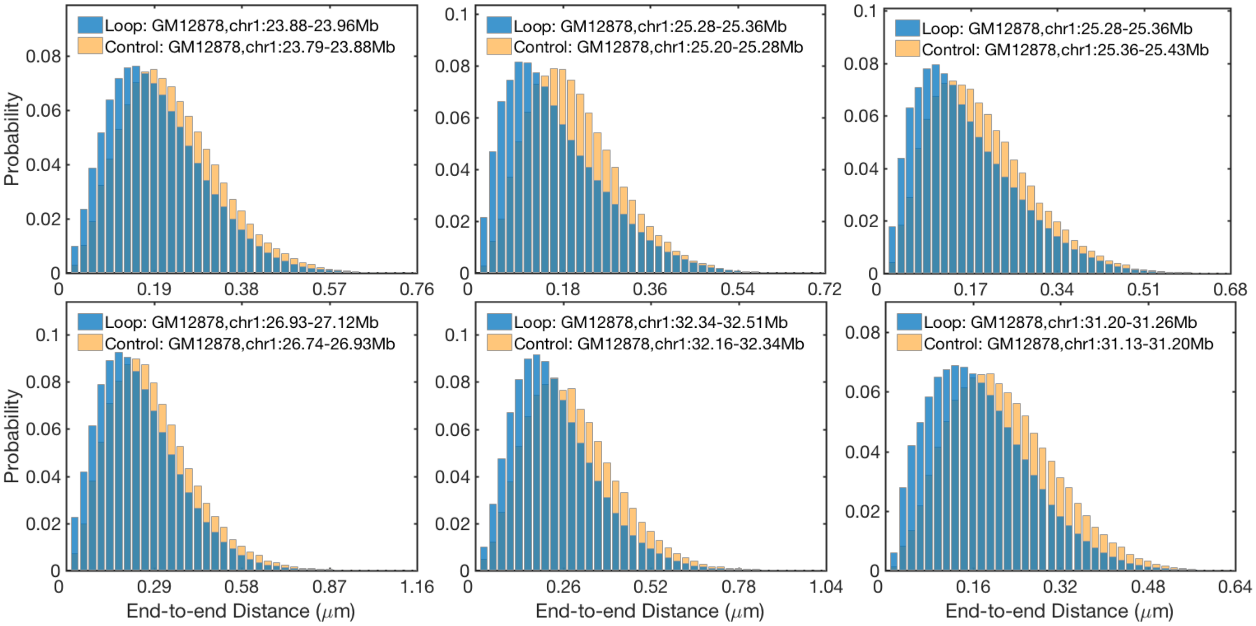

Figure M: Probability distribution of the end-to-end distance for chromatin loops from GM12878 cells (blue). For each chromatin loop, as a comparison, the probability distribution for a control pair with the same genomic distance (yellow) is also shown. The genomic positions for chromatin loops and the corresponding control pairs are indicated in the legend of each plot.

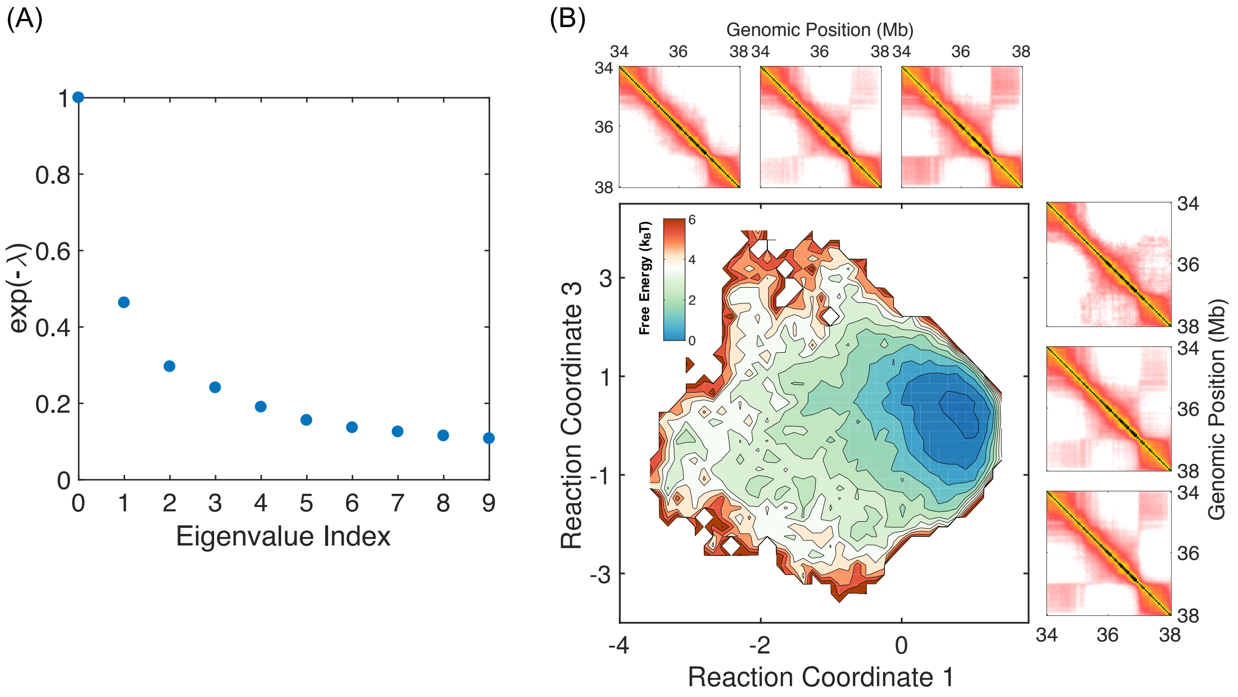

Figure N: (A) Negative exponential of the eigenvalues of the transition matrix calculated in the diffusion map analysis of the genomic region chr1:34-38Mb from GM12878 cells. The eigenvectors corresponding to the second (1) and third (2) eigenvalues are selected to serve as reaction coordinates 1 and 2 respectively. (B) Free energy profile of TAD conformations projected onto eigenfunctions that correspond to the 1st and the 3rd eigenvalues. The (Left) and (Top) panels illustrate the change in contact maps along the two coordinates. The three contact maps for reaction coordinate 1 were identical to those shown in Figure 6, and the three regions used to calculated the contact maps for reaction coordinate 3 are [-3.0,0),[0,1.5),and,[1.5,3.0).

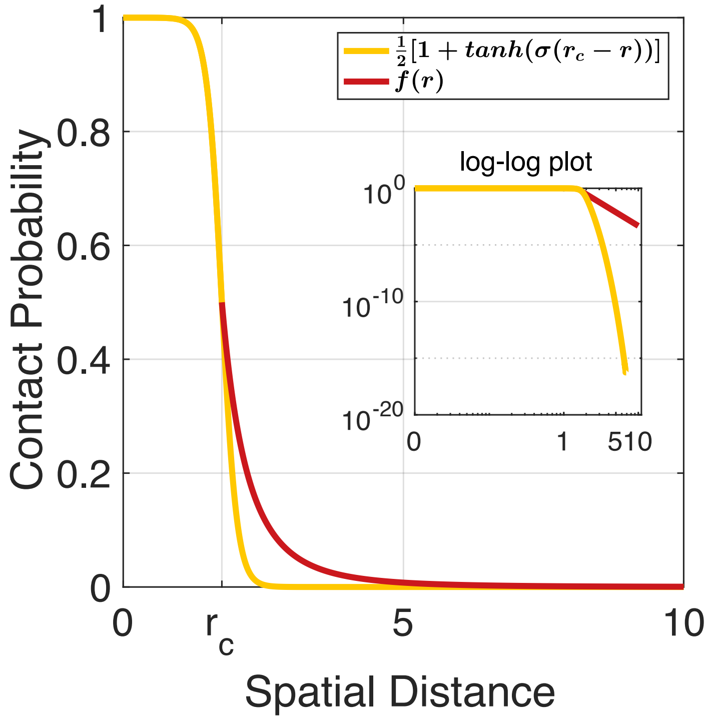

Figure O: The probability of contact formation as measured by the function f(r) defined in Eq. [3] and by a simple switching function. The same plot is shown in the inset on a log-log scale.

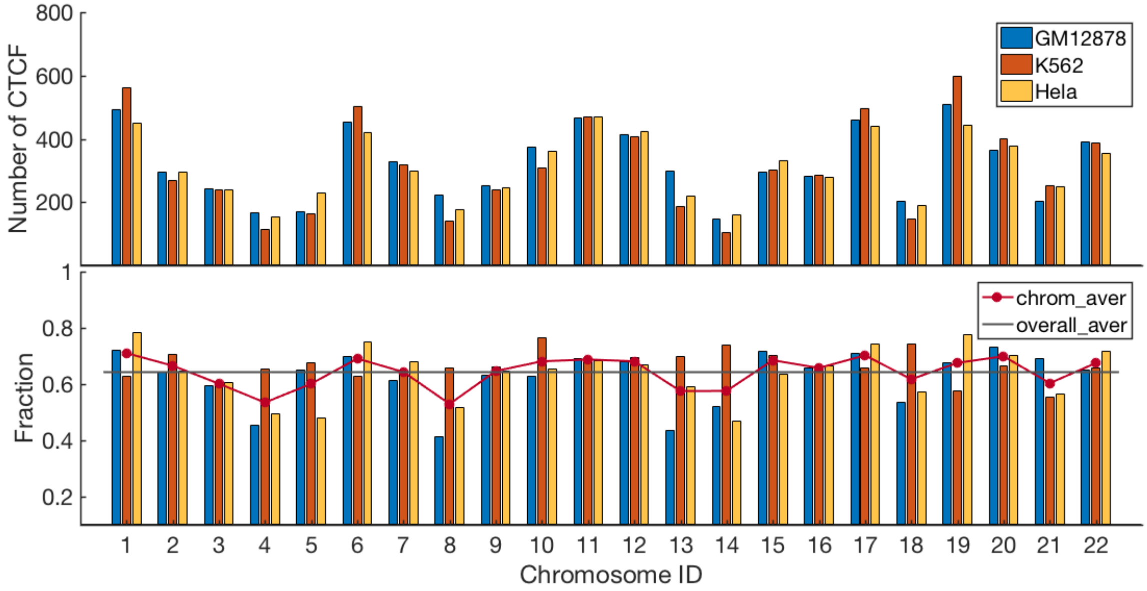

Figure P: **Statistics of CTCF-binding sites for different cell types.** Number of CTCF-binding sites for chromosomes from GM12878 (blue), K562 (orange), and Hela (yellow) cells. (Bottom) The fraction of conserved CTCF-binding sites across the three cell types. The red dots are the aver-age fractions over the three cell types for different chromosomes, and the grey line indicates the average over all chromosomes.
